# Tunable Natural Deep Eutectic Solvents‐Driven Fractionation of Olive Pomace in an Integrated Biorefinery: Linking Lignin Structural Tailoring to Carbohydrate Valorization and Biopolymer Production

**DOI:** 10.1002/cssc.70835

**Published:** 2026-06-27

**Authors:** Ivelina Ivaylova Dzhambazova, Maria Laura Alfieri, Lucia Panzella, Ozge Sakyan Demirkol, Fabio De Stefano, Cinzia Pezzella, Simona Varriale

**Affiliations:** ^1^ Department of Chemical Sciences University of Naples Federico II Naples Italy; ^2^ Department of Food Engineering Ankara University Golbasi Ankara Türkiye

**Keywords:** antioxidants, biomass, deep eutectic solvents, enzymatic saccharification, lignin, polyhydroxyalkanoates

## Abstract

The development of sustainable biorefineries requires integrated fractionation strategies capable of preserving carbohydrate streams while generating high‐value lignin and bioactive fractions. In this study, olive pomace was valorized through a cascade approach combining optimized microwave‐assisted extraction (MAE) of phenolic compounds, natural deep eutectic solvents (NADES)‐enabled pretreatment, and chemo‐enzymatic modification. MAE enabled recovery of a phenolic‐rich fraction and reduced inhibitory compounds in the residual biomass, improving its suitability for downstream processing. The dephenolized substrate was subsequently subjected to NADES‐based fractionation using acidic, alkaline, and neutral systems, including a laccase‐assisted variant. Pretreatment performance depended on solvent chemistry. The acidic system (ChCl:lactic acid) achieved high delignification (≈68%) but produced restructured lignin with extensive β‐O‐4 cleavage. The alkaline NADES (K_2_CO_3_:ethylene glycol) combined high lignin removal (≈75%) with partial preservation of native linkages and solvent‐induced functionalization. The neutral NADES (betaine:glycerol) preserved both polysaccharides and lignin structure, while laccase induced selective oxidative modifications. These differences yielded lignin fractions with distinct structural features suitable for different downstream valorization routes. In parallel, alkaline NADES enabled efficient saccharification with balanced glucose and xylose release, supporting polyhydroxyalkanoate production by *Haloferax mediterranei*. Overall, this work demonstrates a tunable cascade strategy integrating phenolic recovery, DES fractionation, and lignin structural design for multiproduct lignocellulosic biorefineries.

## Graphical abstract

Microwave‐conditioned olive pomace was fractionated with deep eutectic solvents (DESs) spanning acidic, neutral, alkaline, and chemo‐enzymatic regimes. The alkaline DES provided the best balance between delignification and carbohydrate preservation, yielding a fermentable glucose/xylose stream suitable for Haloferax mediterranei fermentation and poly(3‐hydroxybutyrate‐co‐3‐hydroxyvalerate) (PHBV) biosynthesis.

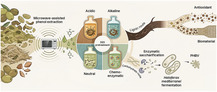

## 1 Introduction

The transition toward a circular and bio‐based economy has intensified interest in biorefineries as integrated systems for the sustainable conversion of renewable biomass into fuels, chemicals, and materials [1, 2, 3]. Modern biorefineries are increasingly designed around cascading valorization schemes, in which different fractions of the same feedstock are sequentially upgraded to maximize carbon efficiency and economic viability. Within this framework, process sustainability is determined not by the yield of a single product but by the ability to generate high‐quality intermediate streams that remain compatible with multiple conversion pathways [4].

Among renewable resources, lignocellulosic biomass represents the most abundant nonfood carbon source available for biorefinery applications [5, 6]. However, its exploitation is fundamentally constrained by intrinsic structural recalcitrance arising from the highly organized architecture of the plant cell wall, where cellulose microfibrils are embedded within a matrix of hemicellulose and lignin [7]. Lignin plays a dual role in this structure: while it provides mechanical strength and resistance to degradation, it also restricts enzymatic accessibility and contributes to the formation of phenolic inhibitors during processing [8]. Consequently, effective biomass valorization requires selective lignin removal while preserving fermentable carbohydrates, particularly chemically labile pentoses. Achieving this balance between delignification and conservation of both C5 and C6 carbon pools remains a central challenge in lignocellulosic biorefinery design [9]. In parallel, growing interest has been directed toward fractionation approaches that enable the recovery of lignin as a structurally and functionally valuable stream rather than as a degraded by‐product [10, 11]. In this regard, so‐called “lignin‐first” strategies have emerged, aiming to prioritize lignin extraction and stabilization during the initial stages of biomass deconstruction [12, 13]. Different chemical pretreatments can generate lignin fractions with distinct molecular architectures, linkage distributions, and reactivity profiles, thereby opening differentiated valorization routes within integrated biorefinery schemes [14, 15].

To address these interconnected requirements—namely, delignification coupled with controlled lignin recovery and carbohydrate preservation—increasing attention has been directed toward solvent‐based pretreatment strategies capable of modulating biomass structure under milder conditions than conventional acid or alkaline processes [16]. In this respect, deep eutectic solvents (DESs) and natural deep eutectic solvents (NADES) have been increasingly explored as versatile and tunable media for lignocellulosic biomass fractionation [17]. Formed through hydrogen‐bond interactions between hydrogen bond donors and acceptors, DES can be rationally formulated to exhibit acidic, neutral, or basic behavior, thereby enabling systematic control over lignin solubilization, carbohydrate stability, and overall deconstruction severity [18]. Recent developments in DES‐based biomass pretreatment have moved beyond conventional binary systems toward more chemically diversified solvent platforms. Acidic binary DESs, including choline chloride‐ or ammonium‐based systems combined with organic acids, have been extensively investigated for promoting lignin solubilization and cellulose accessibility [19]. In parallel, alkaline DESs have emerged as promising alternatives to improve delignification selectivity while limiting hemicellulose degradation [20]. More recently, ternary DESs [21], metal‐assisted DESs [22], and surfactant‐based formulations [23] have been proposed to introduce additional catalytic, Lewis acidic, or amphiphilic functionalities, thereby enhancing lignin disruption, ester/ether bond cleavage, and biomass swelling. However, despite these advances, DES‐based pretreatment studies have largely been evaluated through conventional fractionation metrics, including delignification efficiency, solid recovery, sugar release, enzymatic hydrolysis, and cellulose digestibility [24, 25]. While these parameters are essential, they do not fully describe the suitability of the generated streams for integrated biorefinery applications. In particular, while recent DES studies increasingly address lignin structure or downstream fermentation, fewer reports integrate solvent chemistry, lignin structural fate, mixed C5/C6 carbohydrate preservation, and biological upgrading within the same cascade [26, 27]. In this context, solvent‐driven fractionation alone does not always reconcile these aspects across diverse biomass types. This has motivated the exploration of chemo‐enzymatic strategies, in which DES‐mediated structural deconstruction is combined with selective enzymatic catalysis to further refine lignin architecture without compromising sugar integrity [28]. Herein, laccases (EC 1.10.3.2) represent a particularly relevant enzymatic tool for integration within DES‐based pretreatment strategies. Laccases are multicopper oxidases that catalyze the one‐electron oxidation of phenolic lignin units, generating phenoxyl radicals that may be involved in depolymerization, repolymerization, or structural rearrangement reactions [29, 30]. These transformations can alter lignin molecular weight distribution, interunit linkages, and surface chemistry, potentially affecting lignin extractability, enzyme–lignin interactions, and carbohydrate accessibility [31, 32]. Importantly, several studies have demonstrated that specific neutral deep eutectic solvent systems can maintain or even enhance laccase stability and activity, likely through hydrogen‐bond‐mediated enzyme stabilization and reduced water activity [33, 34]. This compatibility sets the basis for chemo‐enzymatic pretreatment schemes in which DES‐driven lignin disruption is selectively reinforced by enzymatic oxidation, while minimizing sugar degradation by preserving both C5 and C6 carbon pools.

Agro‐industrial residues constitute particularly relevant targets for such selective pretreatment approaches, as they combine high availability with limited competing uses [35]. Within this group, olive pomace (OP) stands out as a chemically complex and challenging lignocellulosic feedstock. Generated in large quantities by the olive oil industry, OP is a humid and heterogeneous residue rich in structural carbohydrates, lignin, residual lipids, and phenolic compounds [36]. Despite its chemical richness, OP is still predominantly managed as a low‐value residue. Its most common destination remains direct combustion for heat and power generation, driven by its residual calorific value [37]. However, this practice is associated with particulate emissions and the release of environmentally harmful compounds, while failing to exploit the intrinsic molecular complexity of the biomass [38]. Such underutilization is largely rooted in the recalcitrant nature of OP. Beyond its lignocellulosic architecture, the biomass contains significant amounts of phenolic compounds that contribute to phytotoxicity and enzymatic inhibition, limiting its direct application in biological conversion platforms [39, 40]. Within emerging biorefinery frameworks, this perception is progressively shifting. Both lignin and low molecular weight phenolic fractions are increasingly recognized as reservoirs of structurally and functionally valuable compounds rather than mere processing barriers [41, 42]. Phenolic compounds constitute one of the most valuable components of olive‐derived residues [43]. Specifically, OP contains a diverse spectrum of bioactive compounds, including phenolic alcohols such as hydroxytyrosol and tyrosol, phenolic acids (e.g., caffeic and *p*‐coumaric acid), flavonoids, lignans, tannins, and complex secoiridoids such as oleuropein and its derivatives [38, 44]. Owing to their antioxidant, anti‐inflammatory, and cardioprotective properties, these molecules have attracted considerable interest for food, nutraceutical, cosmetic, and pharmaceutical applications [43, 45, 46, 47]. Consequently, multiple valorization routes have been explored for their recovery, ranging from conventional solvent extraction [48] to pressurized liquid extraction [49], ultrasound [50], microwave‐assisted extraction (MAE) [51], and supercritical fluid processing [52]. The feasibility of selectively recovering phenolic fractions prior to, or in parallel with, structural biomass fractionation reinforces the positioning of OP as a multiproduct feedstock within integrated biorefinery schemes, rather than a substrate devoted exclusively to C5 and C6 sugar conversion. In such frameworks, economic viability of cascading bioprocessing increasingly relies on the cogeneration of high‐value materials alongside fermentable intermediates [53, 54, 55]. In this regard, polyhydroxyalkanoates (PHAs) represent particularly attractive target products. PHAs are a family of microbial polyesters synthesized intracellularly as carbon and energy storage compounds, combining full biodegradability with tunable physicochemical properties [56]. Their renewable origin and functional versatility position them as promising alternatives to fossil‐derived plastics across a range of applications [57]. From a biorefinery perspective, PHAs offer a distinctive advantage by enabling the direct conversion of carbohydrate‐rich streams into functional materials, thereby maximizing carbon utilization from lignocellulosic feedstocks containing mixed C5 and C6 sugars [58]. The feasibility of producing PHAs from such substrates depends on the availability of microbial platforms capable of coping with substrate complexity and residual process‐derived stressors. Many conventional industrial microorganisms exhibit limited tolerance to inhibitory compounds and inefficient pentose utilization, constraining the valorization of C5‐rich fractions [59]. In contrast, halophilic microorganisms have shown potential as hosts for biorefinery applications due to their intrinsic robustness, tolerance to complex feedstocks, and ability to operate under nonsterile or minimally sterile conditions [60]. Among them, *Haloferax mediterranei* has garnered particular interest as a PHA‐producing archaeon capable of metabolizing a broad range of carbon sources, including pentose sugars [61], and synthesizing poly(3‐hydroxybutyrate‐co‐3‐hydroxyvalerate) (PHBV) without external precursor supplementation [62]. Combined with simplified cultivation and downstream processing under high‐salinity conditions, these features render *H. mediterranei* a suitable platform to assess whether carbohydrate‐rich streams generated from challenging lignocellulosic feedstocks can be biologically upgraded. In this context, PHBV production provides a functional readout of hydrolysate compatibility, complementing the chemical evaluation of phenolic recovery, lignin fractionation, and saccharification performance.

Building on this rationale, OP was explored herein as a multiproduct feedstock in a cascade biorefinery integrating microwave‐assisted phenolic recovery, NADES‐based fractionation, enzymatic saccharification, lignin structural characterization, and PHBV production by *H. mediterranei*. To the best of our knowledge, this is among the first studies coupling DES‐mediated delignification of OP with the biological upgrading of the resulting carbohydrate‐rich stream toward PHAs. Acidic, alkaline, neutral, and laccase‐assisted NADES systems were compared to determine how solvent chemistry governs the balance between delignification, polysaccharide preservation, lignin structural tailoring, and downstream biological compatibility. By connecting pretreatment chemistry with both lignin valorization potential and carbohydrate upgrading, this study moves beyond conventional pretreatment metrics and proposes a stream‐oriented strategy for integrated OP valorization.

## 2 Experimental

### 2.1 Raw Materials and Chemicals

Reagents and materials were purchased from Sigma–Aldrich Corp. (St. Louis, MO, USA), unless otherwise specified. The commercial cellulolytic enzyme cocktail Cellic CTec3 HS (Novozymes A/S, Bagsværd, Denmark), with an enzymatic activity of 102.8 Filter Paper Units (FPU) mL^−1^, was used for enzymatic saccharification experiments. The fungal laccase EV4 (PoxA1b variant) was produced and purified as previously described [33, 63] and used for chemo‐enzymatic pretreatment. *Haloferax mediterranei* DSM 1411 was purchased from the German Collection of Microorganisms and Cell Cultures (DSMZ, Germany). The microorganism was preserved at −80°C in DMSO (5% v/v) as a cryoprotectant agent.

OP was obtained from a local olive oil processing facility in the Ayvalık district of Balıkesir, Türkiye. The biomass was supplied as a single batch in dry form (50 kg sacks) with a moisture content of 7.1%, and no pre‐drying treatment was applied. However, to ensure uniform particle size, the dry samples were ground and sieved through 50‐mesh sieves, resulting in homogeneous particles suitable for extraction experiments. Until the extraction procedures were performed, the samples were stored in amber‐colored bottles under refrigerated conditions at 4°C.

### 2.2 Biomass Compositional Analysis

The chemical composition of the raw biomass and pretreated solid fractions was determined according to the National Renewable Energy Laboratory (NREL) standard protocols. Structural carbohydrates (glucan and hemicellulose) and lignin contents were quantified following the procedure described in NREL/TP‐510‐42618 and NREL/TP‐510‐42622, based on two‐step acid hydrolysis of the biomass. After hydrolysis, the liquid fraction was analyzed by high performance anion exchange chromatography with pulsed amperometric detection (HPAE‐PAD) to quantify the released monomeric sugars. Acid‐soluble lignin (ASL) was determined spectrophotometrically by measuring the absorbance at 320 nm, while acid‐insoluble lignin (AIL) and ash content were determined gravimetrically [64].

### 2.3 Optimization of Phenolic Compounds Extraction

A microwave extraction system (General Electric, GMOM 25, USA) equipped with two Soxhlet apparatuses for condensation and a continuous cooling water flow was used for the extraction process. Samples were placed in an ethanol–water mixture. The solid‐to‐solvent ratio was fixed at 1:20 based on preliminary experiments for a sample volume of 100 mL. The extraction conditions were optimized by using the MINITAB 18 Response optimizer tool for maximum total phenolic content (TPC). The optimum conditions were determined as 270 W, 25 min, and 30% for microwave power, extraction duration, and ethanol percent in solvent, respectively. For the optimum extract, about 0.32 g of extract was obtained per g of starting biomass, corresponding to an extraction yield of around 32%.

### 2.4 TPC Determination and Antioxidant Activity

The TPC was measured using the Folin method, as outlined by Singleton and Rossi [65], with slight modifications. The extracts were mixed with 5 mL of Folin–Ciocalteu reagent and 4 mL of 2 N sodium carbonate, then left to stand at room temperature for 2 h. After incubation, the absorbance was measured at 765 nm. Data are expressed as mg of gallic acid (GA) per mg of extract (gallic acid equivalents, GAE). The antioxidant activity of the extract was evaluated through the 2,2‐diphenyl‐1‐picrylhydrazyl (DPPH) radical scavenging assay as detailed by Alifaki et al. [66] and expressed as mg of DPPH per mg of extract. The exhausted OP biomass resulting from phenol extraction, herein defined as microwave‐conditioned olive pomace (OP‐MW), was then stored at 4°C for further NADES extraction.

### 2.5 HPLC Analysis for Phenolic Profile Determination

Oleuropein, 2,4‐dihydroxyphenyl ethanol, hydroxytyrosol, caffeic acid, ferulic acid, p‐coumaric acid, rutin, gallic acid, chlorogenic acid, tyrosol, vanillic acid, hesperidin, and naringin were examined as the phenolic compounds in the OP extract. The analysis was performed using an Agilent 1100 series HPLC system (USA) equipped with a UV detector and an Eclipse XDB‐C18 column (5 µm, 4.6 × 150 mm). Phenolic compounds were separated using two mobile phases: A (acetic acid:H_2_O, 2:98) and B (ACN:H_2_O, 70:30). The elution gradient was as follows: 95% A/5% B for the initial 3 min, transitioning to 85% A/15% B over 5 min, held for 32 min, followed by 30% A/70% B over 10 min, 10% A/90% B in 1 min, and finally 5% A/95% B for the last 9 min. The flow rate was set at 1 mL/min with a total runtime of 60 min. Readings were taken at 280 nm, with the column temperature maintained at 30°C and an injection volume of 20 µL [66].

### 2.6 DES Preparation and OP‐MW Pretreatment

DES were prepared by mixing the corresponding hydrogen bond acceptor and hydrogen bond donor at the desired molar ratios under magnetic stirring at 80°C until a clear and homogeneous liquid was obtained. The systems investigated in this study were choline chloride:lactic acid (ChCl:LA, 1:4 mol/mol), potassium carbonate:ethylene glycol (K_2_CO_3_:EG, 1:7 mol/mol), and betaine:glycerol (Bet:Gly, 1:2 mol/mol). The prepared DES were stored at room temperature and no crystallization was observed during the period of use.

Preliminary laccase stability assays were carried out to assess enzyme activity in 80% (w/w) Bet:Gly (1:2) DES at 70°C, compared with 20 mM sodium phosphate buffer (pH 7.4), following the method described by Varriale et al. [33], in order to evaluate the suitability of DES conditions for chemo‐enzymatic pretreatment.

Pretreatment experiments were performed using 2 g of dried OP‐MW resulting from the optimized condition of phenol extraction mixed with the corresponding DES according to the solid‐to‐liquid ratio (SLR) of 1:10. The biomass‐DES mixtures were incubated under the conditions summarized in Table 1, including temperature, residence time, and water content. For chemo‐enzymatic pretreatment, the Bet:Gly (1:2) system was combined with the fungal laccase EV4 (10 U g^−1^ biomass). The reaction mixture was initially incubated at 70°C for 1 h to allow enzymatic oxidation, followed by the thermal pretreatment step.

**TABLE 1:** NADES composition and processing conditions for OP‐MW pretreatments.

HBD:HBA, mol/mol	pH	SLR	Temperature, °C	Time, h	H_2_O, % w/w	Reference
Choline chloride:Lactic Acid (1:4)	~1.5	1:10	140	3	20	[67]
Potassium carbonate:Ethylene Glycol (1:7)	~13.0	1:10	130	1	0	[68]
Betaine:Glycerol (1:2)	~6.2	1:10	140	3	20	[28]
Betaine:Glycerol (1:2) + EV4 (10 U g^−1^ biomass)	~6.5	1:10	70 + 140	1 + 3	20	This work

After pretreatment, the recovery of the solid and lignin fractions was performed according to the nature of the DES system and reported in Supporting Information S1. Pretreatment‐induced changes in the morphology and crystalline structure of untreated and treated OP were assessed by scanning electron microscopy (SEM) and X‐ray diffraction (XRD). Experimental details for both analyses, including instrumental settings and calculation of the crystallinity index, are provided in Supporting Information S2.

### 2.7 Two‐Dimensional Heteronuclear Single Quantum Coherence Nuclear Magnetic Resonance (2D‐HSQC NMR) Analysis

The structural features of the recovered lignin fractions were investigated by 2D‐HSQC NMR spectroscopy. Lignin samples (5 mg) were dissolved in deuterated dimethyl sulfoxide (DMSO‐d6) and transferred into 5 mm NMR tubes. Spectra were acquired on a Bruker AVANCE 400 MHz spectrometer (Bruker, Billerica, MA, USA). The residual DMSO signal (δ*H*/δ*C* 2.50/39.6 ppm) was used as an internal reference for chemical shift calibration. The spectra were processed and analyzed using MestReNova software (version 12.0.0, Mestrelab Research) to identify the characteristic ^1^H–^13^C correlation signals of lignin structural units.

### 2.8 Lignin Antioxidant Activity Through DPPH Assay

The antioxidant activity of the recovered lignin fractions was evaluated using the 2,2‐diphenyl‐1‐picrylhydrazyl (DPPH) radical scavenging assay, while the detailed experimental procedure and EC_50_ calculation are provided in the Supporting Information S3.

### 2.9 Enzymatic Saccharification

Enzymatic hydrolysis of the polysaccharide fraction derived from delignified biomass was carried out using the commercial cellulolytic enzyme cocktail Cellic CTec3 (Novozymes). Hydrolysis reactions were performed in 2 mL microcentrifuge tubes with a total working volume of 0.5 mL. Delignified biomass was suspended in 50 mM sodium phosphate buffer (pH 6.0) at the selected solids loading and supplemented with the enzyme cocktail according to the experimental design. For the comparative screening between acidic and alkaline DES‐pretreated biomasses, hydrolysis was performed at 2.5% (w/v) solids loading, corresponding to 12.5 mg dry biomass per reaction, using an enzymatic loading of 60 FPU g^−1^ dry pretreated biomass. This corresponded to a total enzyme input of 0.75 FPU per reaction. For the optimization of K_2_CO_3_:EG‐pretreated biomass saccharification at higher solids loading, the total enzyme input was adjusted either to maintain the same absolute enzyme amount used in the screening assay or to evaluate the effect of increased enzyme dosage. At 5% (w/v) solids loading, corresponding to 25 mg dry biomass per reaction, hydrolysis was carried out at 30 FPU g^−1^ dry pretreated biomass, equivalent to 0.75 FPU per reaction. At 10% (w/v) solids loading, corresponding to 50 mg dry biomass per reaction, hydrolysis was performed at 15 and 30 FPU g^−1^ dry pretreated biomass, corresponding to 0.75 and 1.50 FPU per reaction, respectively. The reaction mixtures were incubated at 50°C under agitation (500 rpm) for 72 h. After hydrolysis, samples were centrifuged (6000 rpm for 10 min), and the supernatants were collected, filtered, and analyzed for monomeric sugars as previously described. The contribution of soluble sugars originating from the enzyme preparation was quantified separately by HPAE‐PAD and subtracted from the measured concentrations to ensure accurate determination of saccharification yields. Saccharification performance was evaluated both as polysaccharide hydrolysis efficiency and as monomeric sugar release. Glucan and xylan hydrolysis efficiencies were calculated from the blank‐corrected glucose and xylose concentrations measured after enzymatic hydrolysis, after conversion into their corresponding anhydro‐polymer equivalents using correction factors of 0.90 and 0.88, respectively:

(1) $$\text{Glucan hydrolysis}\;\text{efficiency}\;\left(\%w/w\right)\;=\frac{C_{\mathrm{Glc}}\;\times \;V\;\times \;0.90}{m_{\mathrm{PS}}\;\times \;X_{\mathrm{PS}}}\;\times \;100$$

(2) $$\text{Xylan hydrolysis efficiency}\;\left(\%w/w\right)\;=\;\frac{C_{\mathrm{Xyl}}\;\times \;V\;\times \;0.88}{m_{\mathrm{PS}}\;\times \;X_{\mathrm{PS}}}\;\times \;100$$

where *C*_Glc_ and *C*_Xyl_ are the glucose and xylose concentrations measured after hydrolysis, expressed as g L^−1^; *V* is the hydrolysis volume, expressed as L; *m*_PS_ is the dry mass of pretreated solid used in the assay, expressed as g; and *G*_PS_ and *X*_PS_ are the glucan and xylan contents of the pretreated solid, expressed as g g^−1^ dry pretreated solid.

Monomeric sugar release was expressed as grams of glucose or xylose released per 100 g of dry pretreated solids. These values were calculated from the glucan or xylan content of the pretreated solid and the corresponding hydrolysis efficiency, using the reciprocal conversion factors:

(3) $$\text{Glucose release }\left(\frac{g}{100\;g_{\text{pretreated solids}}}\right)\;=\;G_{\mathrm{PS},100}\;\times \;\frac{{\mathrm{HE}}_{G}}{100}\;\times \frac{1}{0.90}$$

(4) $$\text{Xylose release}\;\left(\frac{g}{100\;g_{\text{pretreated solids}}}\right)\;=\;X_{\mathrm{PS},100}\;\times \;\frac{{\mathrm{HE}}_{X}}{100}\;\times \;\frac{1}{0.88}$$

where *G*_PS,100_ and *X*_PS,100_ are the glucan and xylan contents of the pretreated solids, expressed as g per 100 g dry pretreated solids, and HE_G_ and HE_X_ are the corresponding hydrolysis efficiencies.

### 2.10 Shake‐Flask Fermentations of *Haloferax mediterranei*

Shake‐flask fermentations were carried out using minimal synthetic medium (MSM) optimized for PHBV production as previously described by Borselleca et al. [69]. The medium composition per liter was: 167.7 g NaCl, 20 g MgSO_4_ · 7H_2_O, 13 g MgCl_2_ · 6H_2_O, 4 g KCl, 0.84 g KNO_3_, 1 g CaCl_2_ · 6H_2_O, 0.5 g KH_2_PO_4_, 0.5 g NaBr, 0.2 g NaHCO_3_, 0.005 g FeCl_3_, and 49.6 mM PIPES buffer. The medium was supplemented with 1 mL L^−1^ SL6 trace element solution and adjusted to pH 6.8–7.0 prior to sterilization. Carbon sources were supplied either as glucose (10 g L^−1^) or as a glucose–xylose mixture of the optimized saccharification hydrolysate. Fermentations were carried out at 37°C and 200 rpm for 72 h. Preliminary growth assays were performed to evaluate the compatibility of saccharification hydrolysates derived from K_2_CO_3_:EG‐pretreated OP‐MW with *H. mediterranei*. Cultures were grown in MSM supplemented either with glucose (2 g L^−1^), xylose (2 g L^−1^), and a mixture of glucose (1 g L^−1^) and xylose (1 g L^−1^) as a control or with an equivalent concentration of sugars provided by the OP‐MW hydrolysate (≈51% glucose, 49% xylose, w/w). Growth was monitored for 72 h.

### 2.11 Polymer Extraction and Characterization

At the end of cultivation, cells were harvested by centrifugation and the polymer extracted from the lyophilized biomass according to Corrado et al. [70]. Polymer recovery was carried out from freeze‐dried cell pellets using chloroform (1 mL per 10 mg of lyophilized biomass) under vigorous stirring overnight. The resulting extracts were clarified by filtration through 0.22 μm PTFE filters, dried under a nitrogen stream, quantified gravimetrically, and stored at −20°C until further analysis. Because of the intrinsically high salt content of *H. mediterranei* biomass, PHBV accumulation was expressed on an ash‐corrected dry cell mass basis. To correct for the residual inorganic content, 200 mg of dry biomass were transferred into a pre‐weighed ceramic crucible and calcined in a muffle furnace at 400°C for 4 h [69]. After cooling to room temperature, the ash residue was gravimetrically determined and subtracted from the initial dry biomass, allowing calculation of the ash‐corrected biomass value. PHBV content was then determined according to Equation (5):

(5) $$\text{PHBV content}\;\left(\%\right)\;=\;\frac{m_{\text{PHBV}}}{m_{\text{dry biomass}}\;-\;m_{\mathrm{ash}}}\;\times \;100$$

where $m_{\text{PHBV}}$ is the recovered polymer mass, $m_{\text{dry biomass}}$ is the initial dry biomass, and $m_{\mathrm{ash}}$ is the residual inorganic fraction after calcination. The monomer composition of the recovered polymers was determined by ^1^H‐NMR spectroscopy. Samples (5–10 mg) were dissolved in 600 μL CDCl_3_ and spectra were recorded on a Bruker DRX‐400 spectrometer (400 MHz). The molar fraction of hydroxyvalerate (HV) was calculated from the integration of the signals corresponding to valerate CH_3_ protons (0.94–0.86 ppm) and butyrate CH_3_ protons (1.33–1.20 ppm) using MestReNova software (version 12.0.0, Mestrelab Research) [69].

### 2.12 Statistical Analysis

Statistical analyses were performed using Minitab 20.4. All experiments were conducted in at least triplicate, and data are reported as mean ± standard deviation (SD). Significant differences among groups were evaluated using Tukey's honestly significant difference (HSD) test at a significance level of *p* < 0.05.

## 3 Results and Discussion

### 3.1 OP Feedstock Characterization

The choice of pretreatment and conversion routes for OP is closely linked to its chemical composition and structural features, which influence biomass recalcitrance and downstream process performance. To this end, OP was characterized to identify key aspects relevant to pretreatment selection according to the standardized NREL protocol [64], and its compositional profile is reported in Table 2.

**TABLE 2:** Chemical composition of untreated OP, determined according to NREL standard methods.

Component	Amount, % w/w
Cellulose	12.0 ± 0.3
Hemicellulose	6.3 ± 0.2
Glucose	13.3 ± 0.1
Xylose	7.04 ± 0.1
Galactose	0.1 ± 0.0
Lignin	42.1 ± 1.0
Acid Insoluble Lignin	40.7 ± 1.0
Acid Soluble Lignin	1.4 ± 0.0
Ash	2.5 ± 0.0
Lipids	0.5 ± 0.1
Phenols (GAE)	0.2 ± 0.1

*Note*: Structural carbohydrates, lignin fractions, ash, lipids, and total phenols are expressed as % (w/w) on a dry biomass basis. Values are reported as mean ± SD.

The biomass exhibits a high lignin content (42.1% w/w), largely associated with the acid‐insoluble fraction (40.7% w/w). This value is consistent with ranges reported in the literature for olive‐derived residues, which are commonly described as lignin‐rich biomasses with limited structural accessibility [38]. The predominance of AIL suggests the possible presence of a condensed lignin network, which contributes to the intrinsic recalcitrance of olive‐derived lignocellulosic residues [71, 72]. Structural carbohydrates are present at moderate levels, with cellulose (glucan) accounting for 12.0% of the dry biomass and hemicellulose mainly represented by xylose (7.0% w/w). Comparable carbohydrate distributions have been reported for OP obtained from different olive oil extraction processes, although absolute values are known to vary depending on cultivar, geographic origin, and processing conditions [38, 44]. The coexistence of fermentable C5 and C6 sugars within a lignin‐rich matrix underscores the potential of OP as a feedstock for biochemical and microbial conversion, while simultaneously emphasizing the need for effective delignification strategies.

In addition to its lignocellulosic components, OP contains inorganic ash (2.5%) and residual lipids (0.5%). Ash contents within this range have been previously associated with reduced enzymatic hydrolysis efficiency and increased variability in bioconversion processes [73, 74]. The phenolic fraction, quantified as GAE, represents a further compositional element of interest. Although present at relatively low concentrations, phenolic compounds are widely recognized as potent inhibitors of enzymatic activity and microbial metabolism, particularly through nonspecific binding interactions with lignin and carbohydrate components of the biomass [75, 76]. However, in a cascade biorefinery perspective, they should not be regarded solely as inhibitory components, but also as a distinct bioactive fraction of potential value. Accordingly, dephenolization can be viewed both as an upstream conditioning step and as an initial valorization stage, aimed at recovering a phenol‐rich stream while improving the suitability of the residual biomass for further fractionation and conversion.

### 3.2 Optimization of Dephenolization Step

MAE was applied as a rapid and efficient dephenolization method, aiming to maximize phenolic recovery while minimizing processing time and solvent consumption. MAE is considered advantageous over conventional extraction approaches due to its ability to promote rapid volumetric heating, enhance cell wall disruption, and improve mass transfer [77]. The extraction conditions were optimized using a Box–Behnken experimental design, where microwave power, ethanol concentration, and process time were selected as independent variables. The TPC values obtained from the experimental runs varied considerably, ranging from approximately 2.41–7.93 mg_GA_ g_extract_^−1^ (Table S1). Such variability indicates that phenolic recovery is highly sensitive to operational conditions and emphasizes the importance of systematic optimization.

The regression model (Equation (6)) developed for TPC prediction confirmed the significant effect of both linear and quadratic terms, highlighting the complex behavior of phenolic extraction under microwave irradiation. The final regression equation indicated significant positive contributions from microwave power, while ethanol concentration exerted a negative linear effect. In practical terms, increasing microwave power enhanced phenolic extraction yield, which can be explained by stronger microwave energy absorption and intensified disruption of plant tissue structure. Higher microwave power likely improved solvent penetration into the matrix and accelerated diffusion of phenolics into the surrounding medium. Similar trends have been reported in MAE studies of phenolic compounds from olive‐derived residues, where microwave power was consistently identified as one of the most influential parameters controlling extraction kinetics [66].

(6) $$Y\;=\;5,205\;+\;0,974X_{1}\;-\;0,702X_{2}\;+\;0,122X_{3}\;+\;0,727{\left(X_{1}\right)}^{2}\;-\;0,232{\left(X_{2}\right)}^{2}\;-\;0,314{\left(X_{3}\right)}^{2}\;+\;1,334\;X_{1}X_{2}\;+\;0,284\;X_{1}X_{3}\;-0,041\;X_{2}X_{3}$$

On the other hand, ethanol concentration showed a negative linear effect, indicating that increasing ethanol percentage beyond a certain threshold may reduce overall TPC recovery. This outcome can be attributed to the diverse polarity of phenolic compounds present in OP. While ethanol–water mixtures are generally effective solvents for phenols, an excessive ethanol content may reduce the extraction of more polar compounds such as hydroxytyrosol derivatives. Furthermore, water content in the solvent system is critical under microwave heating because water has a higher dielectric constant and is more responsive to microwave energy absorption. Therefore, a moderate ethanol concentration may provide an optimal balance between solvent polarity and microwave heating efficiency [78].

The optimization of MAE conditions for phenolic compound recovery from OP was performed by maximizing the TPC using response surface methodology (RSM). The optimum extraction conditions were determined as 270 W microwave power, 25 min extraction time, and 30% ethanol concentration. Under these conditions, the predicted TPC value reached 8.4 mg_GA_ g_extract_^−1^, demonstrating the strong potential of MAE as an efficient dephenolization approach. To confirm the reliability of the optimization, the extraction was carried out again at the selected optimum settings, yielding an experimental TPC of 7.8 mg_GAE_ g_extract_^−1^, which showed good agreement with the predicted result.

In addition to TPC determination, the phenolic profile of the optimized extract was evaluated using HPLC analysis (Table S2). The obtained results revealed that the extract contained a mixture of phenolic acids, flavonoids, and secoiridoid derivatives, confirming that MAE was able to recover a broad range of phenolic compounds. Among the quantified compounds, oleuropein was identified as the main phenolic constituent (0.67 mg g_extract_^−1^), followed by hesperidin (0.53 mg g_extract_^−1^), catechin (0.37 mg g_extract_^−1^), and naringin (0.21 mg g_extract_^−1^). The presence of these compounds is particularly relevant because they are widely associated with strong antioxidant, antimicrobial, and anti‐inflammatory properties [79]. Moreover, the antioxidant activity of optimized extract was also determined as 0.15 mg_DPPH_ g_extract_^−1^. This result is consistent with the phenolic composition of the extract. It also suggests that the MAE process was effective in preserving the antioxidant potential of the extracted compounds.

### 3.3 DES‐Based Pretreatment Strategies for OP Fractionation

DES represent a versatile class of solvents for lignocellulosic biomass pretreatment, as their physicochemical properties can be modulated to influence lignin solubilization and carbohydrate preservation [80]. OP‐MW was therefore used as substrate for DES pretreatment. The compositional analysis of OP‐MW is reported in Table S3. OP‐MW was subjected to pretreatment using DES systems spanning acidic, neutral, and alkaline conditions, including a chemo‐enzymatic variant based on a neutral DES formulation. The DES investigated in this work were selected based on both literature investigation and process rationale. ChCl:LA was included as an acidic benchmark, being one of the most extensively reported DESs for lignocellulosic pretreatment [81]. K_2_CO_3_:EG was chosen as an emerging alkaline alternative reported to better preserve the hemicellulosic fraction while maintaining good delignification performance [68]. Bet:Gly was finally adopted as a neutral comparative system and as the basis for the chemo‐enzymatic variant, owing to the expected compatibility of its betaine‐based formulation with laccase activity [28, 33].

### 3.4 Effect of DES Composition and Chemo‐Enzymatic Integration on Biomass Fractionation

**FIGURE 1:**
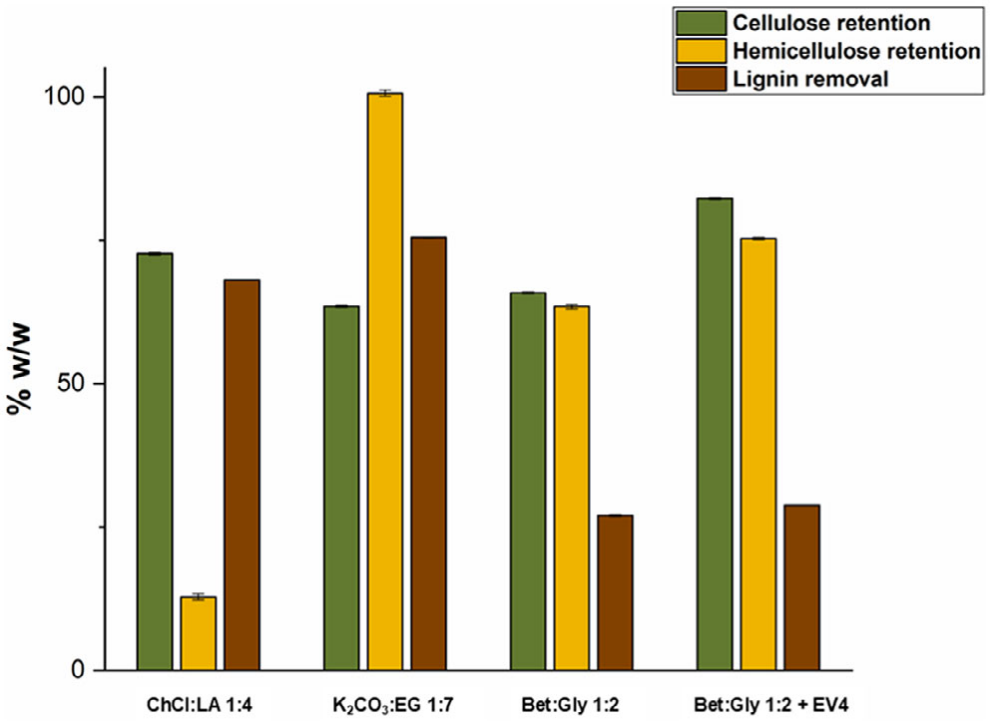
Fractionation performance of the investigated DES systems in terms of cellulose retention, hemicellulose retention, and lignin removal after pretreatment of OP‐MW.The tested conditions included Choline chloride:Lactic Acid (ChCl:LA 1:4 mol/mol), Potassium carbonate:Ethylene glycol (K_2_CO_3_:EG 1:7 mol/mol), Betaine:Glycerol (Bet:Gly 1:2 mol/mol), and Bet:Gly 1:2 combined with laccase EV4. Results are expressed as % w/w relative to the initial biomass composition. Error bars represent SD (*n* = 3).

The effect of DES composition on the fractionation of OP‐MW was evaluated by comparing lignin removal together with cellulose and hemicellulose retention under the different pretreatment conditions (Figure 1), while solid and lignin recovery yields are reported in Table S4.

The results indicate a strong dependence of biomass fractionation performance on DES composition, with acidic, alkaline, and neutral formulations displaying different balances between delignification and the retention of structural carbohydrates.

#### 3.4.1 Acidic DES

The acidic DES ChCl:LA 1:4 mol/mol showed strong delignification capability, reaching 68.1 ± 0.1% lignin removal (Figure 1), in agreement with the behavior commonly reported for choline chloride‐based acidic eutectic systems [82]. Under proton‐rich conditions, acid‐labile linkages within the lignin‐carbohydrate complex, particularly ester and aryl—ether bonds, are readily cleaved, while lactic acid promotes proton‐assisted lignin depolymerization and extraction [83]. In addition, chloride ions contribute to lignin solubilization by disrupting hydrogen–bonding interactions within the lignin matrix and enhancing solvent penetration [84]. However, this high delignification was accompanied by severe hemicellulose loss, with retention decreasing to 12.8 ± 0.5% (Figure 1), consistent with the well‐known susceptibility of xylan to acid‐catalyzed hydrolysis under acidic DES conditions [9]. In lactic acid‐based DES systems, this process is further facilitated by the disruption of hydrogen–bonding interactions within the lignin‐carbohydrate complex, which enhances hemicellulose accessibility to acid‐mediated reactions [85].

Within this framework, the extensive hemicellulose loss observed for the ChCl:LA mixture reflects the broader trade‐off associated with acidic DES pretreatments. While these systems are highly effective in solubilizing lignin, their limited selectivity toward polysaccharide preservation represents a critical drawback when hemicellulose valorization is targeted alongside cellulose recovery.

#### 3.4.2 Alkaline DES

In contrast to the acidic formulation, the alkaline DES K_2_CO_3_:EG 1:7 mol/mol produced a more selective fractionation profile, combining high lignin removal (75.5 ± 0.1%) with substantial retention of cellulose (63.5 ± 0.2%) and quantitative preservation of hemicellulose (Figure 1). Under alkaline conditions, lignin extraction is typically associated with the cleavage of alkali‐labile lignin‐carbohydrate linkages, especially ester bonds [86]. In systems analogous to K_2_CO_3_:EG, lignin separation has been linked in particular to cleavage of the γ‐ester of the lignin‐carbohydrate complex linkages, which detaches lignin from the carbohydrate matrix without extensive disruption of the lignin backbone [68]. This mechanism is consistent with the simultaneous preservation of the hemicellulosic fraction observed here [87, 88].

Ethylene glycol may further contribute by moderating solvent severity, limiting lignin recondensation, and stabilizing carbohydrate‐rich domains through hydrogen–bonding interactions and changes in solvent microenvironment [89]. Under the investigated conditions, the alkaline DES achieved the most favorable balance between delignification and polysaccharide preservation, making it the most selective fractionation system among those tested.

#### 3.4.3 Neutral DES

The neutral DES Bet:Gly 1:2 exhibited a markedly milder pretreatment behavior compared to both acidic and alkaline formulations. High retention of cellulose (65.8 ± 0.2%) and hemicellulose (63.4 ± 0.4%) was observed, while lignin removal remained limited (27.0 ± 0.2%). This pattern reflects the lower severity of neutral DES systems, which lack the strong acidic or basic functionalities required to efficiently disrupt the lignin‐polysaccharide network. The hydrogen‐bonding environment provided by betaine and glycerol likely contributes to carbohydrate stabilization, while mainly inducing biomass swelling and partial loosening of the lignocellulosic matrix rather than extensive delignification [18, 90]. Similar behavior has been reported for other neutral DES formulations, where high carbohydrate preservation is achieved at the expense of low lignin extraction efficiency [89].

#### 3.4.4 Chemo‐Enzymatic Pretreatment

Building on the mild fractionation behavior observed for the neutral Bet:Gly 1:2 system, a chemo‐enzymatic variant integrating fungal laccase EV4 [91] was evaluated under the same solvent conditions. The rationale for this approach was to exploit the relatively enzyme‐compatible environment of neutral DESs while introducing selective oxidative modification of lignin through laccase catalysis. In contrast to acidic or alkaline pretreatments, where lignin removal is primarily driven by chemical solubilization, laccase‐mediated reactions are known to act on phenolic lignin units via one‐electron oxidation mechanisms, potentially inducing both depolymerization and repolymerization phenomena [92]. Under these conditions, the expected effect is not necessarily an increase in bulk lignin solubilization, but rather a modification of lignin architecture that may influence its extractability and the accessibility of the carbohydrate fraction.

Prior to chemo‐enzymatic pretreatment, preliminary activity assays were conducted to assess laccase stability under DES conditions. Residual enzymatic activity was therefore evaluated in 80% w/w Bet:Gly 1:2 and compared with a conventional sodium phosphate buffer at the same temperature of 70°C. As shown in Figure S1, EV4 retained higher residual activity in the DES medium, whereas rapid deactivation was observed in buffer, with activity dropping below 10% after 40 min. By contrast, nearly double residual activity was preserved in the DES system, confirming a stabilizing effect of the solvent environment on enzyme performance.

On this basis, the chemo‐enzymatic protocol was adapted to include an initial incubation step at 70°C for 1 h to accommodate enzymatic oxidation prior to the conventional thermal DES pretreatment at 140°C for 3 h. This configuration allowed laccase catalysis to occur under conditions compatible with enzyme stability while maintaining alignment with the subsequent solvent‐driven fractionation stage.

The chemo‐enzymatic pretreatment resulted in the highest retention of both cellulose (82.3 ± 0.2%) and hemicellulose (75.3 ± 0.2%) among all tested systems, confirming the protective effect of the neutral DES environment on the polysaccharide fraction. In contrast, lignin removal increased only marginally compared to the neutral DES alone (28.8 ± 0.1%), remaining substantially lower than that achieved under acidic or alkaline conditions. This limited increase in lignin extraction suggests that, under the investigated conditions, laccase activity did not primarily promote extensive lignin solubilization. Rather than indicating inefficiency, this behavior is consistent with the well‐documented mode of action of laccases on lignin, which often results in structural rearrangements within the polymeric network rather than net mass removal [93]. Oxidative coupling reactions between lignin radicals can lead to repolymerization or cross‐linking, increasing molecular weight and reducing solubility, even if the chemical functionality and supramolecular organization of lignin are substantially altered [94]. Such transformations may not be captured by gravimetric lignin removal metrics but can nonetheless have profound effects on lignin physicochemical properties, enzyme–lignin interactions, and downstream valorization potential.

In this context, the observed combination of high carbohydrate preservation and moderate lignin modification highlights the distinct role of chemo‐enzymatic pretreatment compared to purely chemical DES systems. While the extent of delignification achieved by Bet:Gly 1:2 + EV4 may be insufficient to significantly enhance enzymatic saccharification under the conditions tested, the laccase‐mediated treatment may have altered lignin accessibility, reactivity, or functional group distribution in ways that are relevant for alternative lignin valorization pathways [95, 96]. The chemo‐enzymatic integration of laccase within a neutral DES system thus delineates a complementary fractionation profile, characterized by extensive polysaccharide preservation and selective lignin modification rather than extensive solubilization. Although this configuration does not match alkaline DESs in terms of delignification efficiency, it introduces a novel pathway that may prove relevant in biorefinery schemes targeting lignin functionalization alongside carbohydrate recovery.

### 3.5 Morphological Effects on Pretreated Biomass

**FIGURE 2:**
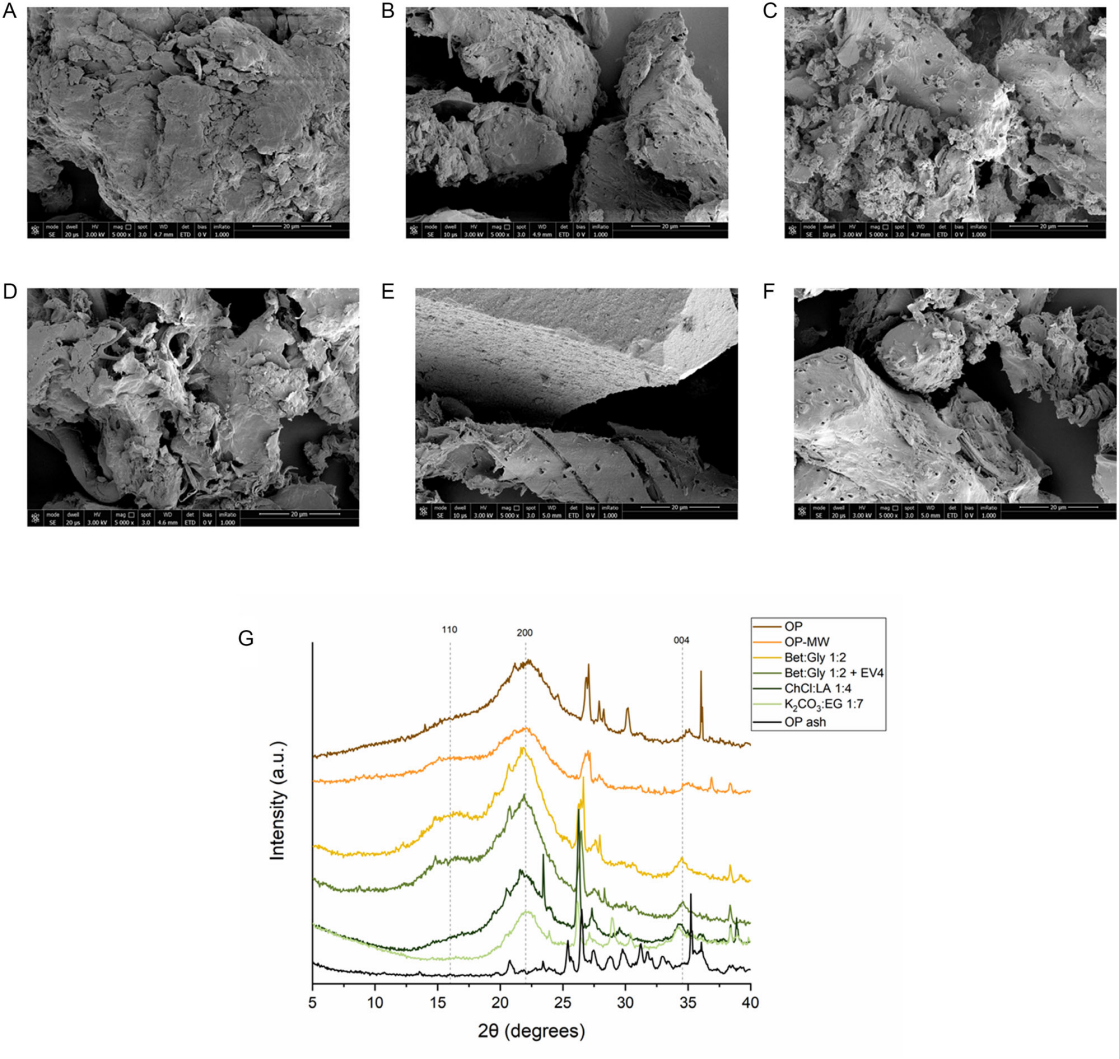
Representative SEM micrographs of untreated OP, microwave‐conditioned OP, and solids recovered after DES pretreatments, acquired at 5000× magnification. (A) untreated OP; (B) OP‐MW; (C) ChCl:LA (1:4)‐treated biomass; (D) K_2_CO_3_:EG (1:7)‐treated biomass; (E) Bet:Gly (1:2)‐treated biomass; (F) Bet:Gly (1:2) + EV4‐treated biomass. (G) XRD patterns recorded in the 2*θ* range 5°–40° for untreated OP, OP‐MW, DES‐pretreated solids, and OP ash. Profiles are shown for Bet:Gly (1:2), Bet:Gly (1:2) + EV4, ChCl:LA (1:4), and K_2_CO_3_:EG (1:7) samples. The main cellulose‐related reflections are indicated.

The structural organization of the solid residues obtained after DES pretreatment was examined by combining SEM and XRD (Figure 2).

SEM observations revealed clear morphological differences among the tested conditions. Untreated OP (Figure 2A) showed dense agglomerates with relatively smooth fracture surfaces, whereas microwave conditioning (Figure 2B) induced only moderate disruption, with localized cracks and cavities. Much more evident morphological changes were observed after ChCl:LA (Figure 2C) and especially K_2_CO_3_:EG pretreatment (Figure 2D), both of which produced irregular, fragmented surfaces with microcavities and exposed fibrous regions, consistent with partial disassembly of the lignocellulosic matrix. Similar features are commonly associated with effective lignocellulosic pretreatments and increased polysaccharide accessibility, although they do not directly predict saccharification performance [97]. By contrast, residues obtained after Bet:Gly treatment (Figure 2E) retained a comparatively compact and cohesive structure, with limited fibrillation and smoother surface domains. The Bet:Gly + EV4 sample (Figure 2F) showed slightly greater heterogeneous morphology than Bet:Gly alone, but still preserved extended compact regions. These features, evidenced by SEM analysis, are consistent with the limited delignification of Bet:Gly‐based systems and may reflect the persistence of a lignin‐rich matrix or the redeposition of lignin‐derived material on biomass surfaces during pretreatment and recovery, as reported for other lignocellulosic systems [98, 99, 100].

XRD patterns of all biomass‐derived samples (Figure 2G) displayed the characteristic broad reflections of cellulose I, centered at 2*θ* values of approximately 16°, 22°–23°, and 34°–35°, with no detectable reflections attributable to cellulose II. This indicates that DES pretreatment altered the relative abundance of biomass components without inducing polymorphic transformation of the crystalline cellulose phase, in agreement with previous observations on olive‐pomace‐derived and other lignocellulosic materials [101, 102]. In addition, narrow reflections at 2*θ* ≈ 25°–32° and 35°–40° were attributed to inorganic crystalline phases, consistent with the diffraction pattern of OP ash and with mineral phases commonly reported in OP ash, including quartz and potassium‐containing salts [103].

The apparent crystallinity index (CrI) increased from ~20%–28% in OP, OP‐MW, and Bet:Gly‐derived residues to ~37% and ~48% in ChCl:LA and K_2_CO_3_:EG samples, respectively. Because CrI in heterogeneous lignocellulosic materials primarily reflects the relative enrichment of cellulose over amorphous components rather than true recrystallization of cellulose domains, these increases are more reasonably interpreted as a consequence of preferential removal of lignin and hemicellulose under acidic and alkaline DES conditions [67, 104]. Conversely, the lower CrI values and compact morphology are evidenced by SEM analysis of the Bet:Gly‐based residues are consistent with the persistence of a larger amorphous fraction, in agreement with their limited fractionation efficiency.

### 3.6 Structural and Functional Characterization of DES‐Extracted Lignin

**FIGURE 3:**
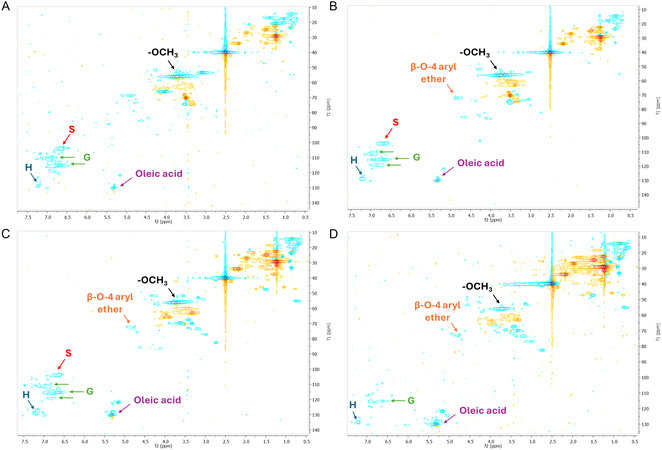
^1^H,^13^C HSQC NMR spectra of the lignin fraction obtained after DES pretreatment with: (A) ChCl:LA 1:4; (B) K_2_CO_3_:EG (1:7); (C) Bet:Gly (1:2) and (D) Bet:Gly (1:2) + EV4.

The structural features of lignins recovered after the different DES pretreatments were investigated by ^1^H and 2D HSQC NMR analysis in order to evaluate the effect of the solvent used on lignin aromatic composition (Figure 3).

As expected for lignin‐like materials, all ^1^H NMR spectra exhibited broad and complex signals in the aromatic region between 6.0 and 7.5 ppm, reflecting the heterogeneous nature of the samples (Figure S2).

For the lignin recovered after the acidic DES pretreatment (Figure 3A), the aromatic region of the ^1^H, ^13^C HSQC spectrum was characterized by the presence of the characteristic correlation peaks C2,6—H2,6 (104/6.6 ppm) of syringyl (S) units together with those due to C5—H5 (115/6.7 ppm) and C2—H2 (111/6.9 ppm) typical of guaiacyl (G) units. Additional signals attributable to *p*‐hydroxyphenyl (H) units were also detected at 127/7.2 ppm. The relative intensity of the correlation peaks indicated a predominance of G‐type structures in this lignin sample. In addition, methoxyl group correlations (56/3.7 ppm) were also clearly detected, indicating the preservation of methoxylated phenylpropanoid structures within the recovered lignin. Notably, however, no detectable correlations attributable to β‐O‐4 aryl ether linkages were observed in the aliphatic region of the spectrum, suggesting the occurrence of extensive cleavage of native ether bonds under acidic conditions, likely leading to lignin depolymerization followed by condensation reactions through the formation of C—C linkages. Such behavior is typical in lignocellulosic biomasses subjected to acidic treatments involving, e.g., lactic acid‐containing DES [23, 105]. It is worth noting that S‐units have been reported to be more readily degraded than G‐units on treatment with acidic DES, resulting in an increased proportion of G‐units in the recovered lignin [23], as indeed observed also in this case (see above). Finally, signals related to residual (free or bound) lactic acid were observed, indicating partial retention of DES‐derived components in the recovered lignin.

The lignin recovered after the alkaline DES treatment (Figure 3B) exhibited a similar pattern of signals in the aromatic region of the ^1^H,^13^C HSQC spectrum, with the presence of S, G, and H units in a ratio similar to that observed for the acidic‐DES derived sample. However, signals attributable to C6—H6 correlations in G units were also detected. In contrast to the acidic lignin, signals compatible with Hα/Cα of β‐O‐4 aryl ether linkages were clearly detected at 73/4.9 ppm. Both these observations indicate that alkaline DES treatment preserved part of the native lignin architecture, without inducing depolymerization/recondensation reactions. The preservation of β‐O‐4 structures is particularly relevant because these ether bonds represent the most abundant and chemically valuable linkages in native lignin and are generally associated with improved lignin processability and valorization potential [106, 107, 108]. However, also in this case, several additional cross‐peaks were detected in the aliphatic region of the spectrum, attributed to ethylene glycol (EG)‐derived moieties. The presence of these signals suggests that, also in this case, the DES not only acted as an extraction medium but also participated in lignin structural modifications, likely through grafting reactions, as previously reported in literature [109].

Moving to the lignin obtained with the neutral DES pretreatment (Figure 3C), the aromatic region of the ^1^H,^13^C HSQC spectrum displayed the expected correlations associated with S, G, and H units similar to those observed for the acid and alkaline lignins, although the signal corresponding to G units C6–H6 correlations appeared less intense. Importantly, signals attributable to β‐O‐4 linkages were clearly detected in this sample, indicating that neutral DES conditions were likewise able to preserve a significant fraction of the native lignin interunit ether linkages. Overall, both alkaline and neutral DES pretreatments were found to be significantly milder than the acidic DES treatment. In contrast, the lignin obtained after treatment with the neutral DES combined with laccase showed a markedly different spectral profile (Figure 3D). The correlation peaks in the aromatic region of the ^1^H,^13^C HSQC spectrum were overall less intense. In particular, the correlations assigned to S units were completely absent, and only those due to C5—H5 of G units and to H units remained detectable. In the aliphatic region of the spectrum, methoxyl group correlations were still observable, whereas signals attributable to β‐O‐4 linkages were significantly less intense compared to the other samples. Altogether, these findings suggest that enzymatic oxidation promoted selective structural modifications of lignin, affecting both aliphatic and aromatic units. In particular, the complete disappearance of S‐unit correlations indicates preferential oxidation of these latter, as expected based on the higher susceptibility to oxidation of S units compared to G and H units. The phenoxy radicals generated during enzymatic treatment may subsequently undergo side‐chain cleavage and radical coupling reactions, ultimately leading to the loss of recognizable S‐unit correlations in the HSQC spectrum.

Interestingly, all analyzed lignin samples exhibited in the ^1^H,^13^C HSQC spectra correlation signals at approximately 130/5.3 ppm, which can be attributed to unsaturated moieties associated with oleic acid residues. Additional intense CH_2_ correlations in the aliphatic region were also detected, which may be attributed to the long aliphatic chains of residual fatty acids or other lipid‐derived compounds naturally present in OP, although overlap with DES‐derived signals cannot be excluded. These observations indicate that none of the DES pretreatments was able to completely remove oleic acid and related compounds naturally present in OP and/or to cleave the ester linkages occurring between these compounds and lignin hydroxyl groups. The persistence of these lipid‐associated structures may influence both the hydrophobicity and the reactivity of the recovered lignins.

Overall, NMR analysis demonstrated that DES composition may strongly influence the structure of the recovered lignin. Acidic DES may promote extensive cleavage of β‐O‐4 ether bonds followed by lignin recondensation, whereas alkaline and neutral DES systems better preserved native interunit ether linkages. In contrast, laccase‐assisted pretreatment induced selective structural modifications in lignin aromatic units, particularly affecting S units. Moreover, the occurrence of solvent grafting phenomena and persistent lignin—lipid bonds highlights the complex reactivity of OP lignin during DES processing. These findings demonstrate that DES composition can be tuned to modulate lignin depolymerization, condensation degree, and functionalization, ultimately affecting the structural properties and valorization potential of the recovered lignin fractions.

### 3.7 Antioxidant Activity and Implications for Lignin Valorization

The antioxidant activity of the recovered lignin fractions was evaluated by the DPPH assay and expressed as EC_50_ values Table 3. In parallel, the TPC of each fraction was determined by the Folin–Ciocalteu assay.

**TABLE 3:** EC_50_ values obtained from the DPPH radical scavenging assay and TPC of lignin fractions recovered after DES pretreatments of OP‐MW.

Pretreatment	EC_50_, µg mL^−1^	TPC, mg_GAE_ mg_Lignin_^−1^
ChCl:LA	366.3 ± 3.7^a^	0.53 ± 0.00^a^
K_2_CO_3_:EG	504.1 ± 13.5^b^	0.38 ± 0.00^b^
Bet:Gly	417.3 ± 20.0^a^	0.33 ± 0.02^c^
Bet:Gly + EV4	723.1 ± 20.5^c^	0.23 ± 0.00^d^

*Note*: Values are reported as mean ± SD. Different lowercase letters indicate significant differences among samples (*p* < 0.05, Tukey's HSD test).

The trend observed for TPC broadly paralleled the antioxidant response, with the acid‐derived lignin showing the lowest EC_50_ and the laccase‐treated fraction the highest one. This overall agreement supports the view that phenolic functionalities are major contributors to DPPH radical scavenging in lignin systems, in line with literature showing that antioxidant activity generally increases with phenolic hydroxyl content and, to a lesser extent, with methoxyl substitution [110]. However, the present results also indicate that phenolic abundance alone does not fully determine antioxidant performance. This is particularly evident in the comparison between the alkaline and neutral lignins: although the K_2_CO_3_:EG fraction exhibited a higher TPC than the Bet:Gly lignin, its antioxidant activity was lower. This discrepancy indicates that DPPH scavenging may depend not only on the amount of phenolic groups, but also on their structural context, accessibility, and effective reactivity within the lignin matrix. More generally, lignin antioxidant behavior is known to be influenced by a combination of phenolic hydroxyl content, molecular weight, degree of condensation, and accessibility of reactive aromatic moieties in solution [111, 112]. Within this framework, the ChCl:LA lignin appears to combine the most favorable features for radical scavenging. HSQC analysis identified this fraction as the most structurally reorganized, as indicated by the disappearance of detectable β‐O‐4 correlations, while methoxylated aromatic units remained preserved. Although these data do not directly quantify phenolic hydroxyl groups, they are fully consistent with a lignin fraction in which substantial ether‐bond cleavage and aromatic reorganization have generated a more accessible redox‐active network. In other words, the superior DPPH activity of the acid‐derived lignin is plausibly associated with the simultaneous occurrence of phenolic enrichment and pronounced structural deconstruction, which together favor the interaction between lignin antioxidant sites and the radical probe. By contrast, the Bet:Gly lignin showed an antioxidant activity close to that of the acidic fraction despite an HSQC profile characterized by preservation of β‐O‐4 linkages and retention of the main S/G/H aromatic units. This result is particularly informative because it shows that the neutral DES appears to preserve a lignin architecture in which antioxidant‐active aromatic motifs remain sufficiently exposed and chemically available, even in the absence of marked backbone cleavage. Thus, the comparison between ChCl:LA and Bet:Gly suggests that high radical scavenging capacity may arise from structurally different lignin states: one strongly restructured and likely enriched in accessible phenolic sites, the other more preserved but still functionally reactive. A different situation was observed for the K_2_CO_3_:EG lignin. Lignins recovered with similar alkaline DES systems have been reported to display strong antioxidant properties when enriched in phenolic hydroxyl groups and G/H‐type structures [67]. However, the present OP‐derived lignin did not follow this pattern, despite its relatively high TPC. HSQC showed partial preservation of the native lignin architecture together with the presence of EG‐related cross‐peaks, indicating solvent participation in lignin modification. A plausible interpretation is that these solvent‐derived modifications did not enhance antioxidant reactivity and may instead have altered the local environment of phenolic groups, reducing their effective accessibility in the DPPH assay. Since radical scavenging in this system depends primarily on phenolic hydrogen‐donating groups rather than on additional aliphatic hydroxyl functionalities, the apparent EG grafting is not expected to directly improve antioxidant performance. Finally, the Bet:Gly + EV4 lignin displayed both the lowest TPC and the weakest antioxidant activity, and this trend was fully coherent with its distinct HSQC profile. The attenuation of aromatic correlations, disappearance of syringyl‐associated signals, and lower intensity of β‐O‐4 cross‐peaks point to a more extensive oxidative remodeling of the lignin network. This behavior is consistent with the known action of laccases, which oxidize phenolic lignin units to phenoxy radicals that may subsequently undergo coupling and repolymerization, thereby reducing the pool of readily available phenolic hydrogen‐donating groups. Although laccase treatment can in some cases enhance lignin antioxidant properties, its effect is strongly substrate‐ and condition‐dependent [113]; in the present case, the chemo‐enzymatic treatment appears to have shifted the lignin toward a more condensed and less redox‐accessible structural state.

These observations are particularly relevant from a lignin‐first perspective because they indicate that pretreatment chemistry determines the balance between lignin depolymerization, structural preservation, and chemical functionalization. In other words, the choice of DES does not merely control how much lignin is removed from the biomass, but also defines the molecular properties of the lignin stream that becomes available for downstream use. Lignins obtained under acidic conditions, being more extensively restructured, may be better suited for routes based on fragmentation, depolymerization, or the generation of low‐molecular‐weight aromatic fractions [114]. Conversely, lignins recovered under alkaline and neutral conditions, which retain a greater proportion of native ether linkages and aromatic integrity, may represent more suitable substrates for applications where controlled structural preservation is desirable, such as the development of lignin‐based functional materials [10, 95]. More broadly, the results support the idea that DES pretreatment can be regarded not only as a biomass deconstruction step, but also as a tool for tuning lignin product quality within integrated biorefinery schemes. Under this view, lignin ceases to be interpreted solely as a recalcitrant matrix component to be removed in order to access sugars, and instead emerges as a coproduct whose structural features can be modulated according to the intended valorization route.

### 3.8 Enzymatic Saccharification of DES‐Pretreated Solids

Enzymatic saccharification assays were first conducted to evaluate the hydrolytic digestibility of all pretreated OP biomasses. Preliminary trials performed on Bet:Gly and Bet:Gly + EV4 substrates yielded poorly reproducible hydrolysis profiles (data not shown), most likely due to their elevated residual lignin content. High lignin levels are well known to hinder enzymatic hydrolysis through multiple inhibitory mechanisms. Residual lignin can physically limit enzyme accessibility to cellulose microfibrils, while dissolved lignin fractions and other phenolic compounds may further impair cellulase stability and activity [7, 115, 116, 117]. In addition, lignin can promote nonproductive adsorption of cellulolytic enzymes via hydrophobic, electrostatic, and hydrogen–bonding interactions, effectively reducing the pool of catalytically available enzymes [118]. Such nonspecific binding phenomena are recognized as a major factor limiting hydrolysis efficiency in lignin‐rich substrates [119]. Furthermore, solubilized lignin fragments may reprecipitate onto biomass surfaces during hydrolysis, forming droplets or redeposited layers that further obstruct enzyme access to structural carbohydrates [120].

**FIGURE 4:**
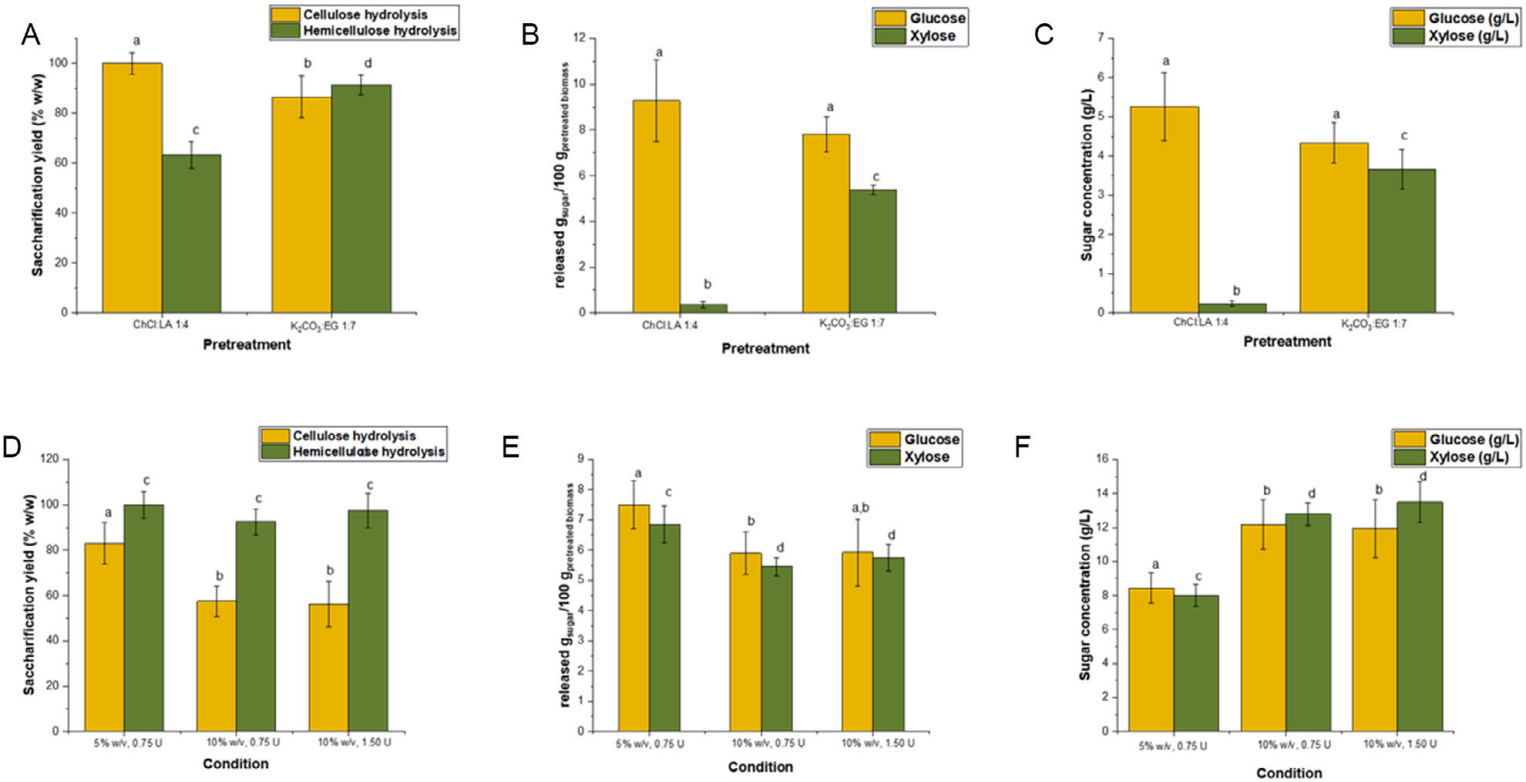
Saccharification performance of DES‐pretreated OP‐MW. (A–C) Comparison between acidic and alkaline DES pretreatments: (A) cellulose and hemicellulose conversion yields (% w/w); (B) released glucose and xylose expressed as g per 100 g of pretreated biomass; (C) glucose and xylose concentrations in hydrolysates (g L^−1^). (D–F) Effect of solids loading (5 and 10% w/v) and enzyme dosage (0.75 and 1.50 U) on the saccharification of K_2_CO_3_:EG‐pretreated OP‐MW: (D) cellulose and hemicellulose conversion yields (%w/w); (E) released glucose and xylose expressed as g *per* 100 g of pretreated biomass; (F) glucose and xylose concentrations in hydrolysates (g L^−1^). Error bars represent SD (*n* = 3). Different lowercase letters indicate significant differences within each panel (*p* < 0.05, Tukey's HSD test).

Taken together, these lignin‐mediated inhibition effects likely contributed to the low reproducibility and limited saccharification performance observed for the neutral DES‐pretreated biomasses, where lignin removal remained insufficient to alleviate enzymatic accessibility constraints. In contrast, substrates obtained through ChCl:LA and K_2_CO_3_:EG pretreatments, characterized by substantially higher delignification efficiencies, were expected to provide a more hydrolytically accessible carbohydrate matrix. These biomasses were therefore selected for comparative saccharification screening. Screening hydrolysis assays were conducted at 2.5% (w/v) solids loading using a fixed Cellic Ctec3 dosage of 0.75 FPU, and performance was evaluated in terms of both saccharification yield and released sugars *per* 100 g of pretreated biomass, as shown in Figure 4A–C.

Biomass pretreated with ChCl:LA exhibited the highest cellulose digestibility, reaching complete glucan conversion and the highest glucose release (9.30 ± 1.80 g per 100 g pretreated biomass; Figure 4B). This behavior is consistent with the strong delignification and extensive hemicellulose solubilization induced by the acidic DES, which increases cellulose accessibility by removing major structural barriers to enzymatic attack. However, this high glucan conversion was accompanied by limited xylan hydrolysis (63.3 ± 5.4%; Figure 4A) and only marginal xylose release (0.35 ± 0.10 g per 100 g biomass; Figure 4B), reflecting the substantial depletion of the hemicellulosic fraction during pretreatment. As a result, the hydrolysate generated under acidic conditions was largely dominated by glucose.

A different pattern was observed for the K_2_CO_3_:EG system, which produced a more balanced saccharification profile. Although glucan conversion was moderately lower (86.6 ± 8.5%), hemicellulose hydrolysis was markedly higher (91.4 ± 4.1%; Figure 4A), resulting in a substantially greater xylose release (5.39 ± 0.20 g per 100 g biomass; Figure 4B). This behavior is consistent with the combined effect of efficient delignification and preservation of the hemicellulosic fraction under alkaline DES conditions. These contrasting outcomes confirm that pretreatment chemistry governs not only the extent of enzymatic digestibility, but also the composition of the resulting sugar stream: the acidic DES maximized cellulose conversion, whereas the alkaline DES enabled simultaneous recovery of both hexose and pentose fractions.

When contextualized within the OP literature, the saccharification performances obtained here are fully consistent with, and in some respects superior to, previously reported values. Organosolv‐pretreated OP hydrolyzed with Cellic CTec2 has been reported to reach glucan conversions of approximately 70% [36]. Likewise, dilute‐acid and liquid‐hot‐water pretreatments of OP have achieved glucan hydrolysis yields approaching 90%–95%, but often at the expense of extensive hemicellulose degradation and poor pentose recovery [121]. In this respect, the ChCl:LA system closely resembles conventional acid‐based fractionation, whereas the more balanced behavior of K_2_CO_3_:EG aligns with the growing interest in pretreatment strategies capable of preserving both carbohydrate pools [122]. This feature is particularly relevant for downstream fermentation and multiproduct biorefinery schemes, where the co‐release of glucose and xylose enhances feedstock versatility. In addition, DES‐based fractionation proceeds under milder and more environmentally compatible solvent conditions than conventional organosolv or mineral‐acid pretreatments [6].

On this basis, K_2_CO_3_:EG was selected for further process optimization, in order to improve hydrolysate sugar titers while preserving its favorable mixed‐sugar profile. The effect of increasing biomass loading (5 and 10% w/v) and enzyme dosage on saccharification performance is shown in Figure 4D–F.

At 5% (w/v) solids loading, saccharification proceeded with high overall efficiency, reaching complete hemicellulose conversion and 83.0 ± 9.1% cellulose hydrolysis (Figure 4D). Under these conditions, glucose and xylose were released in comparable amounts, confirming the good accessibility of both polysaccharide fractions after alkaline DES pretreatment (Figure 4C). Increasing biomass loading to 10% (w/v) at the same enzyme dosage led, as expected, to higher absolute sugar concentrations in the hydrolysate (Figure 4F), but this gain was accompanied by a pronounced reduction in saccharification yields, particularly for cellulose, which dropped to 57.5 ± 6.7% (Figure 4D). This behavior is consistent with the onset of enzyme limitation and mass‐transfer constraints commonly observed at elevated solids loadings, where increased viscosity and reduced mixing efficiency hinder enzyme diffusion and substrate accessibility [123, 124].

Doubling the enzyme dosage at 10% (w/v) only partially alleviated these limitations and did not restore conversion yields to the levels observed at lower solids loading (Figure 4F), indicating that the reduced hydrolysis efficiency was not due to enzyme dosage alone but also to physicochemical constraints intrinsic to high‐solids systems [125]. The 5% (w/v) condition therefore represented the most favorable balance between conversion efficiency and hydrolysate concentration, combining high saccharification yields with a balanced glucose/xylose output. For this reason, hydrolysates obtained under these conditions were selected for the subsequent fermentation assays.

### 3.9 Fermentation of Mixed C5/C6 Hydrolysates by Haloferax mediterranei and PHBV Production

The utilization of lignocellulosic hydrolysates as fermentation feedstocks is often hindered by the potential formation of inhibitory compounds during pretreatment, such as furfural, 5‐hydroxymethylfurfural (HMF), weak organic acids, and phenolic derivatives, which can impair microbial growth and product yields [75]. For halophilic archaea such as *H. mediterranei*, the tolerance toward such inhibitors remains poorly characterized compared to conventional bacterial hosts. Therefore, the compatibility of the enzymatic hydrolysate with *H. mediterranei* was first evaluated through preliminary growth assays. Since this stream contained both glucose‐ and xylose‐derived sugars, its physiological accessibility was first examined by comparing *H. mediterranei* growth on defined carbon sources and on the OP‐derived saccharified hydrolysate at the same total sugar concentration of 2 g L^−1^. As shown in Figure S3, glucose and the glucose/xylose mixture supported rapid growth over 72 h, whereas xylose as the sole carbon source resulted in a markedly attenuated profile. The OP‐derived hydrolysate showed a slower initial increase but progressively reached final OD_600_ values comparable to those observed with the glucose‐containing conditions. This behavior suggests that the real hydrolysate matrix may introduce a transient substrate‐ or process‐related effect, without causing a persistent limitation of *H. mediterranei* growth at the tested concentration. The comparison with the defined glucose/xylose control is relevant because both conditions supplied mixed C5/C6 sugars, whereas only the hydrolysate contained the residual matrix generated during pretreatment and enzymatic saccharification. Thus, the convergence of the hydrolysate culture toward comparable final growth provides a functional indication of hydrolysate compatibility. At the same time, the poor performance observed on xylose alone indicates that pentose assimilation represents a less favorable physiological condition for *H. mediterranei* than glucose‐containing media, consistent with the greater complexity of archaeal pentose metabolism [126]. Having established the suitability of the OP‐derived hydrolysate as a growth‐supporting substrate, the stream was then evaluated under the PHBV‐accumulating regime previously optimized for *H. mediterranei* in terms of salinity, nitrogen source, and C/N ratio [69]. This 72 h cultivation was therefore designed to test the transfer of an optimized polymer‐accumulating condition to a process‐derived hydrolysate. The reference MSM condition contained glucose as the sole carbon source at 10 g L^−1^, whereas the OP hydrolysate condition supplied a mixed C5/C6 carbon stream corresponding to 8.4 g L^−1^ glucose and 8.1 g L^−1^ xylose. The resulting CDW and PHBV accumulation are reported in Table 4.

**TABLE 4:** Cell dry weight (CDW) and PHBV accumulation of *H. mediterranei* cultivated in shake flasks for 72 h under the reference MSM condition and under the OP hydrolysate.

Condition	**CDW, g L** ^**−1**^	**PHBV, % g**_**polymer**_ **g**_**CDW**_^**−1**^
MSM [69]	6.87 ± 0.35^a^	24.82 ± 3.16^a^
OP hydrolysate	7.89 ± 0.54^b^	20.62 ± 1.05^a^

*Note*: PHBV content is expressed as % (g_polymer_ g_CDW_^−1^). Values are reported as mean ± SD. Different lowercase letters indicate significant differences between conditions (*p* < 0.05, Tukey's HSD test).

Under the hydrolysate condition, PHBV accumulation remained in the same order of magnitude as that observed in the reference MSM condition, reaching 20.6 ± 1.0% (g_polymer_ g_CDW_^−1^) compared with 24.8 ± 3.2% in the glucose‐based medium. This decrease in polymer content did not parallel a reduction in biomass formation, suggesting that the hydrolysate mainly affected the partitioning of assimilated carbon toward storage metabolism rather than the overall capacity of *H. mediterranei* to grow on the process‐derived stream. This behavior is consistent with the recognized metabolic versatility of the microorganism, which has been repeatedly described as a robust PHBV‐producing archaeon capable of growth and polymer accumulation on complex and waste‐derived substrates, including hydrolysate‐based carbon sources [127, 128, 129].

From a metabolic perspective, the lower PHBV accumulation observed under the OP hydrolysate may reflect a less favorable allocation of carbon toward storage polymer synthesis relative to glucose‐only cultivation. In *H. mediterranei*, PHBV biosynthesis is tightly linked to central carbon metabolism, and the nature of the carbon source can influence not only growth but also the partitioning of carbon toward biomass formation, maintenance, and polymer accumulation [130]. In this context, the simultaneous presence of glucose and xylose likely imposed an additional metabolic adjustment compared with glucose‐only feeding, resulting in a modest decrease in polymer storage without compromising the overall feasibility of the process. This aspect is relevant from a bioprocess perspective, since lignocellulosic‐derived sugar streams are intrinsically more heterogeneous than defined glucose media, and one of the major challenges in their valorization is precisely the ability to maintain polymer accumulation under mixed and compositionally variable lignocellulose‐derived carbon sources [131]. Accordingly, the endpoint comparison indicates that the optimized PHBV‐accumulating regime can be transferred to a real OP‐derived carbon stream, while a more detailed resolution of substrate hierarchy, sugar uptake dynamics, and possible matrix effects will require dedicated kinetic profiling of hydrolysate‐fed cultivations.

The structural identity of the recovered polymers further supports this conclusion. As shown in Figure S4, the ^1^H‐NMR spectra obtained under the hydrolysate‐profile condition were fully consistent with the PHBV polymer.

Likewise, the HV molar fraction corresponded to 12% mol/mol, indicating that the mixed‐sugar feeding regime did not markedly affect copolymer composition, when compared to the statistically optimized condition reported in Borselleca et al. [69] This point is particularly relevant because the physicochemical and mechanical properties of PHBV are strongly dependent on monomer distribution [132], and the HV contents measured here fall within the range commonly reported in the literature for *H. mediterranei*‐derived PHBV [62]. The limited variation observed therefore, indicates that the OP hydrolysate allowed both PHBV accumulation and the biosynthesis of a structurally comparable copolymer. Taken together, these results support the technical feasibility of using a C5/C6 sugar stream for *H. mediterranei* fermentation. Although the reference MSM condition remained slightly more favorable in terms of PHBV accumulation, the preservation of polymer production within the same order of magnitude, together with the maintenance of HV content, supports the integration of OP fractionation and haloarchaeal biopolymer production within a lignocellulosic biorefinery framework.

### 3.10 Positioning of the Proposed Cascade Within OP Biorefineries

**FIGURE 5:**
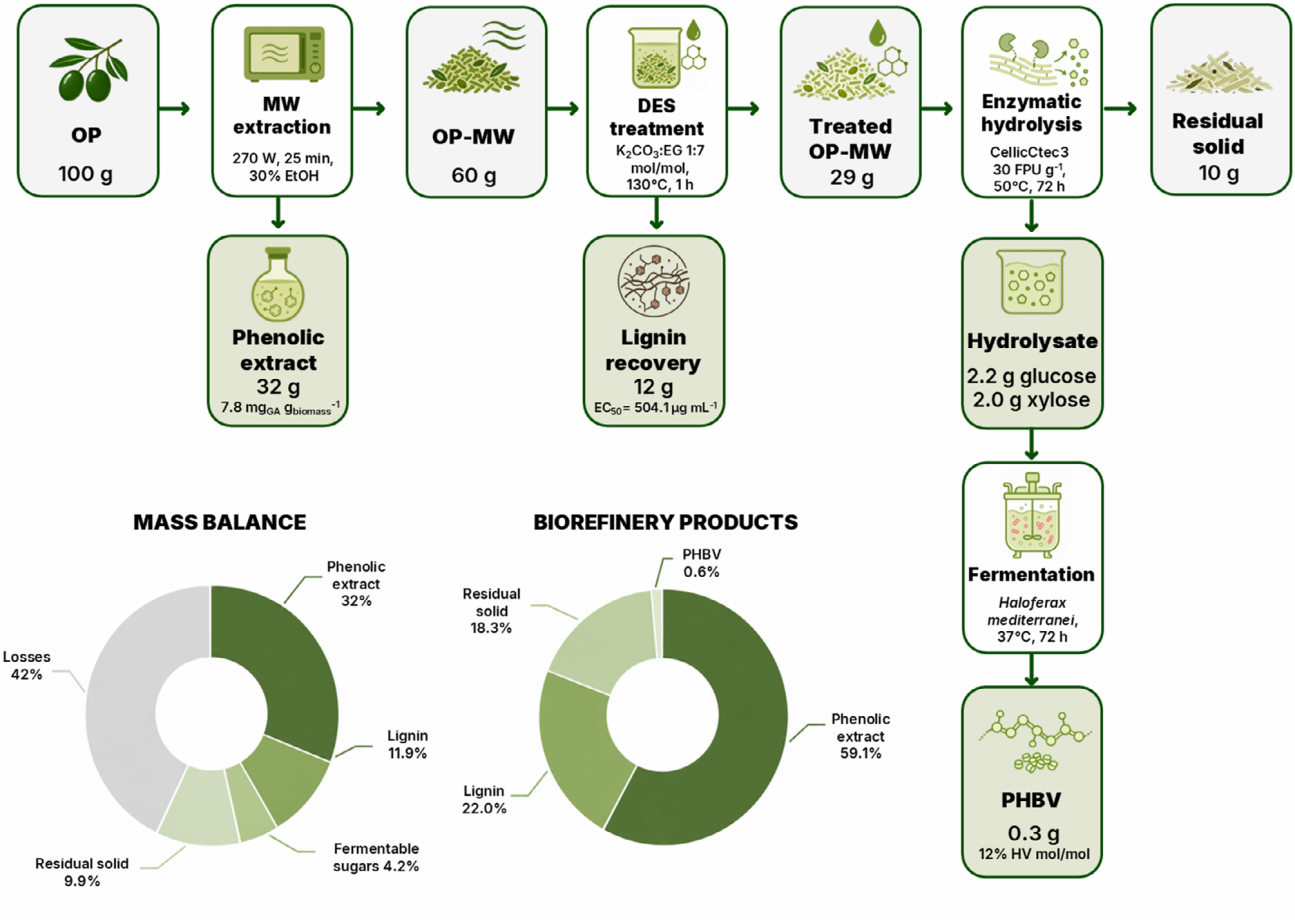
Mass balance of the proposed integrated OP upgrading route.

The overall mass balance shown in Figure 5 provides a quantitative framework to position the proposed process within recently reported OP biorefineries. In the process described by Nicodemou et al. [133], 100 g of OP generated a water‐extract liquid containing 9.1 g sugars and 1.2 g polyphenols, followed by a dilute‐acid liquid fraction containing 33.2 g sugars and 2.0 g polyphenols, and an enzymatic hydrolysate containing 12.2 g sugars, while the final residual solid remained highly enriched in lignin (24.8 g). Similarly, Manzanares et al. [121] reported that 100 kg of exhausted OP yielded 35.6 kg of water extract containing 8.0 kg glucose and 5.0 kg phenolic compounds, 27.9 kg of prehydrolysate enriched in hemicellulosic sugars, particularly xylose (5.5 kg), and an enzymatic hydrolysate containing 7.0 kg glucose and 1.0 kg xylose, together with a final solid residue largely composed of lignin. A related, but more integrated, approach was proposed by Gómez‐Cruz et al. [36], in which 100 g of exhausted OP were sequentially converted into an aqueous extract containing 4.5 g mannitol and 4.5 g phenols, an organosolv lignin fraction (4.2 g), and an enzymatic hydrolysate containing 7.4 g glucose. Taken together, these studies show that OP can be effectively fractionated into phenolic‐rich extracts, sugar streams, and lignin‐rich fractions.

These examples provide an important benchmark, as they show that OP can be effectively partitioned into multiple streams. However, most of these schemes remain primarily centered on carbohydrate mobilization, with lignin either recovered as a separate downstream fraction or retained in the final solid residue. Against this background, the present process should not be interpreted as a direct attempt to outperform sugar‐oriented pretreatments. Indeed, the overall fermentable sugar recovery was lower than that reported for some dilute‐acid or organosolv‐based OP biorefineries. This represents a current limitation of the cascade, particularly with respect to carbohydrate conversion efficiency. Nevertheless, the lower sugar output also reflects a different process rationale: the pretreatment was designed to redistribute the main biomass constituents into chemically and biologically exploitable streams, rather than to maximize the release of a single hydrolysate fraction. The alkaline K_2_CO_3_:EG treatment is particularly relevant in this respect, as it enabled extensive lignin removal while retaining a carbohydrate‐rich solid suitable for enzymatic saccharification. This configuration introduces a lignin‐oriented fractionation step upstream of hydrolysis, thereby avoiding the treatment of lignin solely as a recalcitrant residue remaining after sugar extraction. At the same time, the preservation of both glucose‐ and xylose‐derived fractions allowed the resulting hydrolysate to be incorporated into the biological branch of the cascade through *H. mediterranei* cultivation. Although PHBV production was demonstrated only as a preliminary endpoint experiment, the maintenance of polymer accumulation from the olive‐pomace‐derived mixed‐sugar stream supports the compatibility of this fraction with halophilic bioconversion. A further element distinguishing this study from conventional pretreatment comparisons is the inclusion of chemically divergent NADES environments. In particular, the laccase‐assisted Bet:Gly condition adds a chemo‐enzymatic dimension to this framework, showing that enzyme‐compatible NADES media can operate as reactive environments for selective lignin modification. Although this route was not the most favorable for sugar release, its relevance lies in the possibility of modulating lignin structure under comparatively mild and carbohydrate‐preserving conditions, thereby broadening the range of lignin‐centered outputs accessible from the same feedstock. A further process‐level aspect concerns the fate of the solvent phase after pretreatment. The use of K_2_CO_3_:EG is compatible with recovery strategies reported for related alkaline DES systems [68], in which spent liquors can be processed through lignin precipitation, salt removal, solvent regeneration, and pH readjustment prior to reuse. However, in the present OP matrix, the composition of the recovered DES phase was not experimentally resolved, and solvent recycling was not directly assessed. This point is particularly relevant because spent NADES liquors may contain dissolved lignin fragments, residual sugars or oligosaccharides, inorganic salts, ash‐derived components, and extractive‐derived impurities, all of which could affect solvent performance over repeated cycles [134]. Therefore, a complete evaluation of the cascade will require matrix‐specific analysis of impurity build‐up in the recovered solvent phase, together with reuse experiments aimed at determining solvent losses, regeneration efficiency, and the persistence of pretreatment performance.

On this basis, the mass balance should be interpreted as defining the current process window of the cascade rather than as a final yield‐optimized configuration. The present layout does not yet maximize carbohydrate recovery and does not establish solvent circularity at the process scale. However, it identifies the main trade‐offs governing further development: lignin removal versus carbohydrate preservation, stream diversification versus fermentable sugar yield, and solvent functionality versus regeneration requirements. Within this framework, the improvement of sugar recovery, the validation of DES regeneration, and the assessment of impurity build‐up in recycled solvent phases emerge as the critical steps for consolidating the cascade beyond the present proof‐of‐concept stage.

## 4 Conclusion

This work demonstrates that OP can be effectively valorized through an integrated cascade strategy combining phenolic extraction, NADES‐based fractionation, and chemo‐enzymatic processing. The preliminary removal of phenolic compounds via optimized MAE enabled the recovery of a high‐value bioactive fraction, while improving the processability of the residual biomass by reducing inhibitory effects.

NADES pretreatment proved to be highly tunable, with solvent chemistry governing the balance between delignification, carbohydrate preservation, and lignin structural modification. Acidic systems promoted extensive lignin depolymerization and β‐O‐4 cleavage, whereas alkaline NADES enabled efficient lignin removal while partially preserving native linkages and introducing solvent‐derived functionalities. In contrast, neutral NADES conditions maximized polysaccharide retention and maintained lignin structural integrity, while laccase‐assisted treatment selectively modified lignin aromatic units. From a process perspective, the proposed cascade enabled the recovery of multiple product streams from the same feedstock, including phenolic extracts, lignin fractions with distinct structural features, and fermentable sugars. Based on the overall mass balance, approximately 32% of the initial biomass was recovered as phenolic‐rich extracts, 12% as isolated lignin, and 4.2% as fermentable sugars available for bioconversion, corresponding to the recovery of the 48.2% of the starting feedstock.

All the obtained outputs represent marketable products, attractive also for the pharmaceutical and material sectors. In this regard, the efficient saccharification of alkaline‐pretreated biomass and the successful conversion of mixed C5/C6 sugars into polyhydroxyalkanoates demonstrate the compatibility of the process with downstream bioconversion into added‐value products.

Overall, this work highlights the potential of NADES‐based fractionation to maximize carbon utilization and support multiproduct biorefinery configurations.

## Funding

This work was supported by European Union Partnership for Research and Innovation in the Mediterranean Area (PRIMA) (P2P/PRIMA/0123/0062).

## Conflicts of Interest

The authors declare no conflicts of interest.

## Supporting information

The authors have cited additional references within the Supporting Information.

## Data Availability

The data that support the findings of this study are available from the corresponding author upon reasonable request.
