# Supplementary material for: Tunable Natural Deep Eutectic Solvents‐Driven Fractionation of Olive Pomace in an Integrated Biorefinery: Linking Lignin Structural Tailoring to Carbohydrate Valorization and Biopolymer Production
Source: ChemSusChem. 2026 Jun 27;19(13):e70835. doi: 10.1002/cssc.70835 (PMC13309918; doi:10.1002/cssc.70835)
Supplement: Supplementary file 1 — The authors have cited additional references within the Supporting Information. [file CSSC-19-e70835-s001.pdf]

Supplementary information S1: Recovery of solid and lignin fraction after pretreament

For the alkaline K_2CO_3:EG pretreatment, the reaction mixture was cooled to 50°C and 100 mL of ethanol solution (50%, v/v) and 100 mL of hot water were added to facilitate filtration. The solid fraction was separated by filtration and washed with distilled water until neutral pH to remove residual DES and soluble components. The liquid fraction was acidified to pH 2 using 1 M HCl, followed by dilution with 1000 mL of distilled water to induce lignin precipitation. Both the recovered solid fraction and the precipitated lignin were washed with distilled water until neutral pH and subsequently freeze-dried for further characterization.[59] For the acidic and neutral systems (ChCl:LA, Bet:Gly, and Bet:Gly + EV4), the solid fraction was separated by centrifugation and washed with ethanol followed by deionized water to remove residual solvent. Ethanol washing fractions were collected and the solvent was removed by rotary evaporation. Lignin was then precipitated from the concentrated liquor by adding ten volumes of 0.01 M HCl, recovered by centrifugation, washed with distilled water until neutral pH, and freeze-dried prior to gravimetric quantification and further characterization.[61]

Solid and lignin recovery, cellulose retention, hemicellulose retention, and lignin removal were calculated based on the compositional analysis of the pretreated solids according to the following equations. The compositional fractions used in the calculations were normalized to the initial composition of OP-MW.

$$Solid\ Recovery\ (SR)\ (\%\ w/w)\ =\frac{m_{residual}}{m_{initial}}\ \times\ 100\ (Equation\ S1.1)$$

$$Cellulose\ retention\ (\%\ w/w)\ =\frac{C_{cellulose,residual}\times SR}{C_{cellulose,raw}}\times\ 100\ (Equation\ S1.2)$$

$$Hemicellulose\ retention\ (\%\ w/w)\ =\frac{C_{hemicellulose,residual}\times SR}{C_{hemicellulose,raw}}\times\ 100\ (Equation\ S1.3)$$

$$Lignin\ removal\ (\%\ w/w)\ =\left(1-\ \frac{C_{lignin,residual}\times SR}{C_{lignin,raw}}\right)\times\ 100\ (Equation\ S1.4)$$

$$Lignin\ recovery\ (\%\ w/w)\ =\left(\frac{Extracted\ lignin}{C_{lignin,raw}}\right)\times\ 100\ (Equation\ S1.5)$$

where $m_{initial}$ is the initial dry mass of OP-MW before pretreatment and $m_{residual}$ is the dry mass of the recovered solid fraction after pretreatment. $C_{component,raw}$ and $C_{component,residual}$ represent the compositional fraction (%) of cellulose, hemicellulose, and lignin in the raw OP-MW biomass and in the pretreated solids, respectively, as determined by NREL compositional analysis.

Supplementary Information S2: Morphological characterization of untreated and treated OP

The morphology of untreated and pretreated olive pomace solids was examined using field emission scanning electron microscopy (FE-SEM, FEI Nova NanoSEM 450). Solid samples were mounted on aluminum stubs using double-sided carbon tape and sputter-coated with a thin layer (approximately 5–7 nm) of gold–palladium alloy prior to analysis.

SEM images were acquired at an accelerating voltage of 5 kV using secondary electron detection.

The crystalline structure of the biomass samples was analyzed by X-ray diffraction (XRD). Diffraction patterns were recorded at room temperature using Ni-filtered Cu Kα radiation (λ=0.15418 nm). Powder diffraction profiles were collected using an Empyrean diffractometer (Malvern Panalytical) operating in reflection geometry, with continuous scans over the 2θ range of 5-40° at a scanning rate of 0.02 °/s.

The crystallinity index (CrI) of the samples was calculated according to the Segal method.[120]

$$CrI\ (\%) = \frac{I_{002} - I_{am}}{I_{002}} \times 100 \quad (Equation\ S2.1)$$

where I_{002} corresponds to the intensity at ~22°, while I_{am} identifies the amorphous regions at ~18°.

Supplementary Information S3: Lignin antioxidant activity through DPPH assay

Lignin samples were initially prepared as stock solutions (1 mg mL^{-1}) in DMSO and subsequently diluted in ethanol to obtain a range of concentrations for half-maximal effective concentration (EC_{50}) determination.

For the assay, 100 µL of each lignin dilution were mixed with 900 µL of a methanolic DPPH solution (0.05 mg mL^{-1}) and incubated for 30 min in the dark at room temperature. Methanol was used as the blank, while a mixture of 900 µL DPPH solution and 100 µL methanol served as the control. The absorbance was measured at 517 nm using a UV–Vis spectrophotometer. All measurements were performed in triplicate. The radical scavenging activity of the lignin samples was calculated according to the following equation:

$$Radical\ Scavenging\ activity\ (\%) = \frac{A_{control} - A_{sample}}{A_{control}} \times 100 \quad (Equation\ S3.1)$$

where $A_{control}$ is the absorbance of the control solution and A_{sample} is the absorbance measured in the presence of the lignin sample.

The EC_{50} was determined by plotting the radical scavenging activity (%) as a function of lignin concentration and fitting the resulting curve. Curve fitting and EC_{50} determination were performed using OriginPro® 2018 software. The EC_{50} value corresponds to the concentration of lignin required to achieve 50% inhibition of the DPPH radical.

Table S1. Box Behnken experimental design microwave extraction and TPC of olive pomace extracts

MW power (W)	Ethanol percent (%)	Process time (min)	TPC ($mg_{GA}\ g_{extract}^{-1}$)
-1	0	-1	5.0
-1	-1	0	2.6
1	-1	0	7.9
0	1	1	3.2
1	1	0	3.1
0	-1	1	5.0
0	0	0	5.2
0	0	0	5.2
0	1	-1	2.4
0	0	0	5.2
1	0	-1	6.6
1	0	1	6.8
-1	1	0	5.1
0	-1	-1	4.0
-1	0	1	4.0

Table S2. Concentrations of phenolic compounds in the extracts.

Phenolic compound	Standard curve equation	Extract Phenolic content (mg $g_{extract}^{-1}$)
Gallic acid	y = 59.449x + 11.404	0.2033±0.0067
Hydroxytyrosol	y = 22.729x + 3.3712	0.1038±0.0329
Cateshin	y = 13.074x – 18.631	0.3747±0.0041
Chlorogenic acid	y = 22.288x – 0.7062	0.0422±0.0087
Tyrosol	y = 12.081x – 1.1021	ND
Vanilic acid	y = 33.826x – 7.0911	ND
Cafeic acid	y = 56.968x + 8.6631	0.0701±0.0083
p-Coumaric acid	y = 91.972x – 4.7971	0.0633±0.0038
Rutin	y = 10.951x – 2.4513	ND
Ferulic acid	y = 42.357x – 9.518	0.0541±0.0019
Naringin	y = 28.437x – 15.266	0.2148±0.0470
Hesperidin	y = 9.9221x + 14.717	0.5283±0.0050
Oleuprein	y = 6.4056x – 3.8004	0.6692±0.1368

ND:not detected

Table S3. Compositional analysis of OP-MW expressed as % (w/w) on dry biomass basis. Values are reported as mean ± standard deviation.

Component	Amount (% w/w)
Cellulose	14.1 ± 2.2
Hemicellulose	5.9 ± 1.2
Glucose	15.7 ± 2.2
Xylose	6.7 ± 1.2
Lignin	38.9 ± 1.4
Acid Insoluble Lignin	37.5 ± 1.4
Acid Soluble Lignin	1.4 ± 0.0
ASH	1.3 ± 0.3

Table S4. Solid e lignin recovery yields (SR e LR, respectively) of pretreated OP-MW expressed as % (w/w). Values are reported as mean ± standard deviation.

Pretreatment	SR (% w/w)	LR (% w/w)
Bet:Gly + EV4	74.8 ± 2.5	20.1 ± 1.2
Bet:Gly	70.4 ± 5.2	36.3 ± 2.9
K_2CO_3:EG	44.0 ± 1.2	51.2 ± 0.4
ChCl:LA	38.7 ± 5.6	46.7 ± 3.1

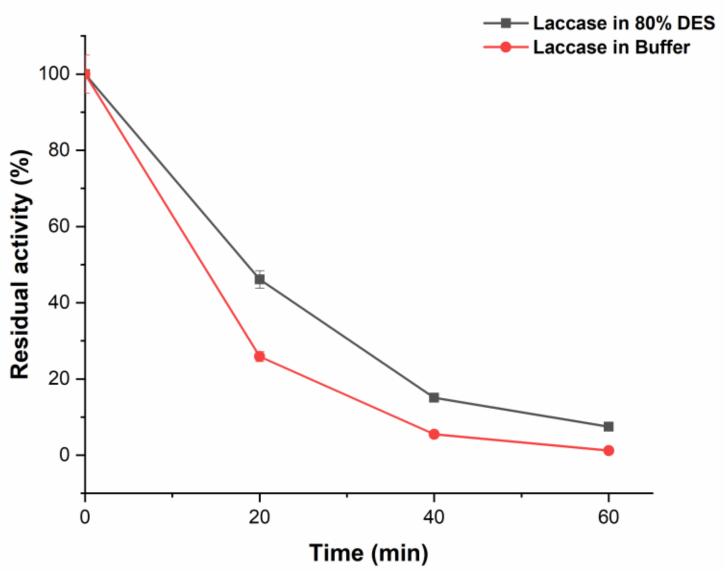

Figure S1. EV4 laccase activity in either 80% w/w Betaine:Glycerol 1:2 mol/mol DES or in 20 mM sodium phosphate buffer at pH 7.4, measured at different timeframes after incubation at 70°C.

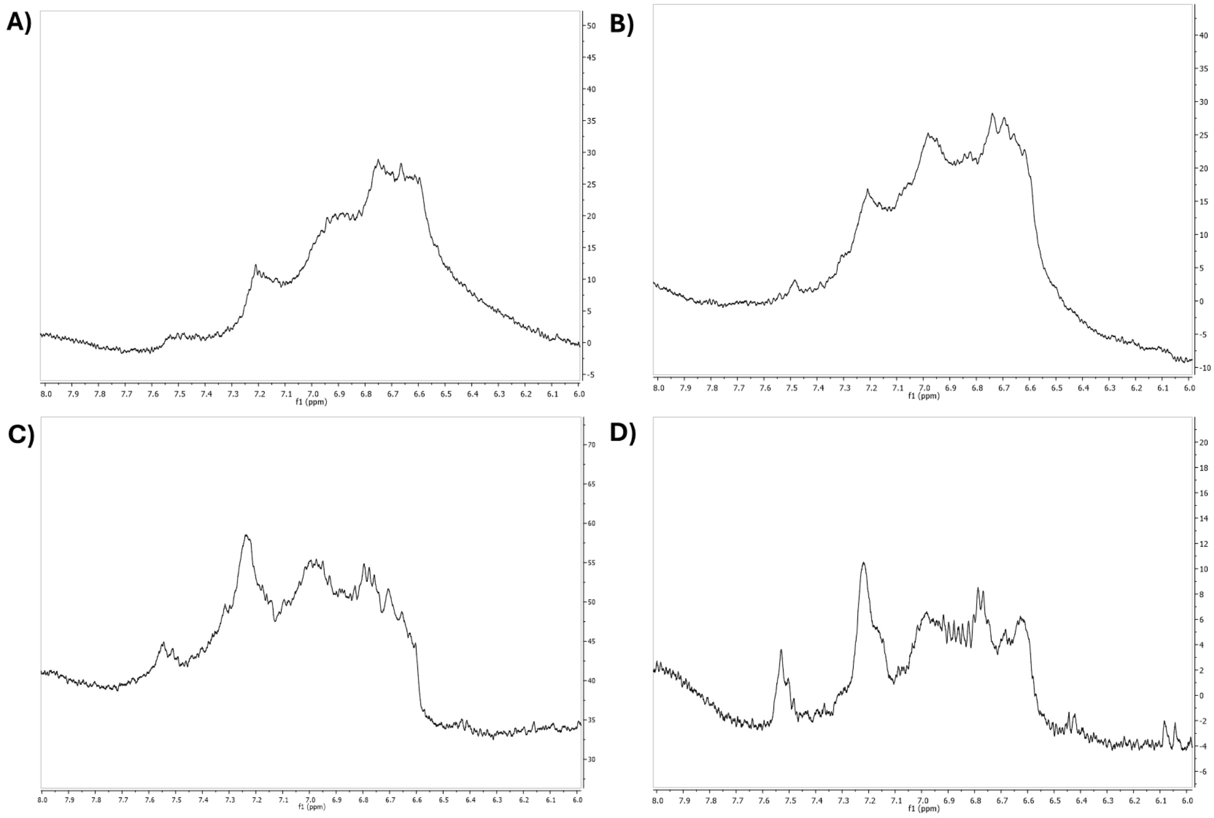

Figure S2. ^{1}H NMR spectra of the lignin fraction obtained after DES pretreatment with: (A) ChCl:LA 1:4; (B) K_2CO_3:EG (1:7); (C) Bet:Gly (1:2) and (D) Bet:Gly (1:2) + EV4.

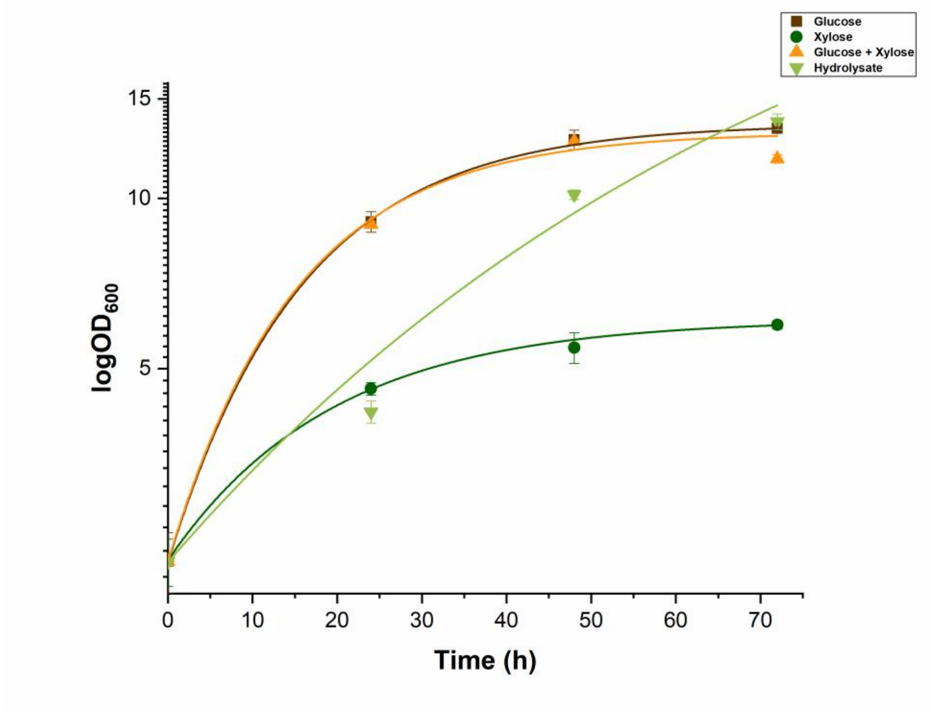

Figure S3. Growth profiles over the course of 72 hours of *H. mediterranei* cultivated in MSM supplemented with glucose, xylose, glucose/xylose, or OP-derived saccharified hydrolysate as carbon sources. All conditions were adjusted to the same total sugar concentration of 2 g L^{-1}. The mixed-sugar control contained glucose and xylose at 1 g L^{-1} each, while the hydrolysate condition provided an equivalent total sugar concentration from the K_2CO_3:EG-pretreated OP-MW saccharification stream. Data are reported as mean ± standard deviation.

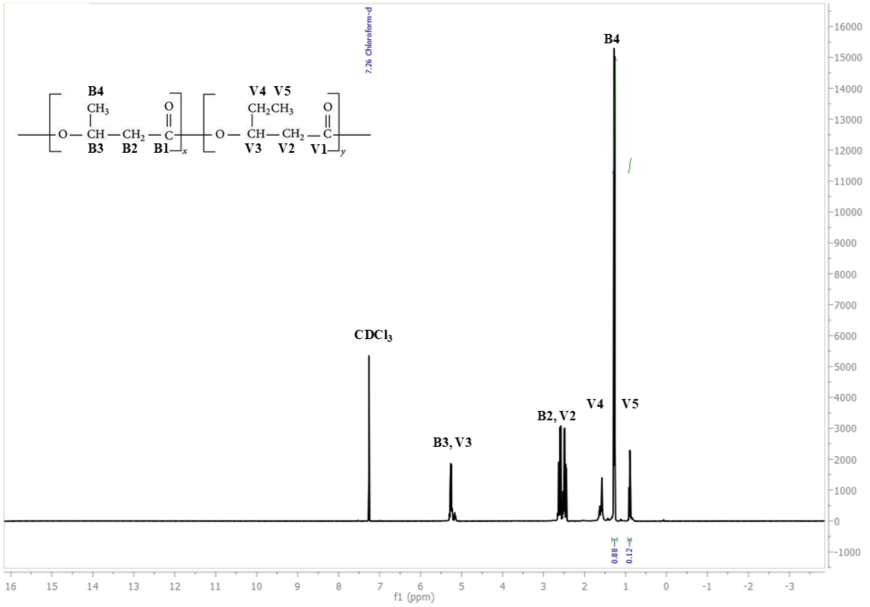

Figure S4. ^{1}H-NMR spectrum of the polymer recovered from *H. mediterranei* lyophilized biomass after chloroform extraction. 3HB unit: B2, methylene (CH_2); B3, methine (CH); B4, methyl (CH_3). 3HV unit: V2, methylene (CH_2); V3, methine (CH); V4, methylene (CH_2); V5, methyl (CH_3).
